# Ligand Topology Tunes Energy and Character of Emissive Triplet States in Stannylenes

**DOI:** 10.1021/acs.inorgchem.6c01229

**Published:** 2026-05-28

**Authors:** Philipp Sikora, Robert Naumann, Katja Heinze, Christoph Förster

**Affiliations:** Department of Chemistry, 9182 Johannes Gutenberg University Mainz, Duesbergweg 10−14, 55128 Mainz, Germany

## Abstract

In the last decades, phosphorescent or photoactive complexes with earth-abundant transition metals have gained huge attention. On the contrary, heavy main group elements with similar properties are rare, in particular group 14 complexes in the oxidation state + II, e.g. tetrylenes. The excited state properties have remained mostly undiscussed and general design concepts for excited state properties—as established for transition metal complexes—are lacking. The present study offers a conceptional topological approach for understanding and addressing specific excited state properties of main group complexes, exemplary discussed by the Sn­(^*t*−Bu^pdp^*t*–Bu^) complex **1** with the dianionic pincer-type ligand [^*t*−Bu^pdp^*t*–Bu^]^2–^ (H_2_^*t*–Bu^pdp^*t*–Bu^ = 2,6-bis­(3,5-di-*tert*-butylpyrrol-2-yl)­pyridine). **1** shows red triplet ligand-to-metal charge transfer (^3^LMCT) phosphorescence in the solid state. Emission intensities of solid **1** vary in an unusual manner with temperature. The obtained experimental results are underpinned and interpreted with detailed (SOC-TD)­DFT calculations. The investigation of **1** in solution was prevented by formation of a bridged dimer **2**. The present results are put into context with the few reported studies on the photophysics of stannylenes to achieve a deeper general understanding of the photophysics of this class of complexes.

## Graphical abstract

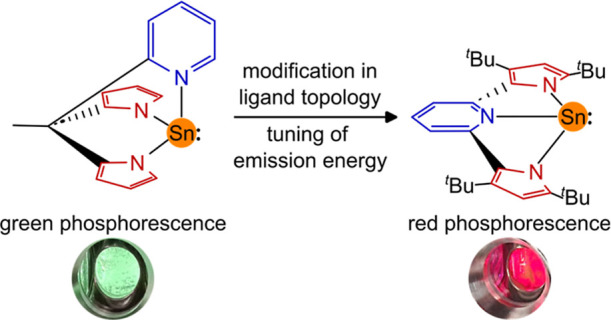

## Introduction

Photoluminescence of transition metal complexes, in particular photoactive transition metal complexes (TMCs) of earth-abundant elements, mainly 3d metal ions, developed into a viable alternative to complexes with precious transition metals like ruthenium, iridium, platinum or gold.^1–11^ The prerequisite for photochemical applications is a sufficiently long lifetime of the excited states (ESs), which is often realized for ESs with different spin multiplicities than the ground state (GS), leading to spin-forbidden nonradiative and radiative processes between ES and GS, consequently extending the ES lifetime.^12^ Alternatively, an ES of different spin multiplicity can serve as a reservoir for a fluorescent state which is repopulated in the thermally activated delayed fluorescence (TADF) cascade, thus extending the ES lifetimes, a phenomenon frequently observed in copper­(I) complexes.^13–17^ In all cases, nonradiative processes need to be mitigated. Clear design concepts and structure property relationships were developed for TMCs: (i) the emissive/photoactive ES should be rather undistorted, i.e. nested, either directly by the character of the ES, e.g. doublet spin-flip and triplet metal-to-ligand charge transfer (^3^MLCT) states in chromium­(III) and iron­(II) complexes, respectively,^4,6,7,9^ or by rigidification, e.g. in case of pseudotetrahedral copper­(I) complexes preventing the flattening distortion.^13–17^ (ii) Detrimental (distorted) ESs opening pathways of nonradiative deactivation should be energetically separated from the emissive/photoactive states, e.g. the separation of the metal-centered (MC) states from the envisaged photoactive ^3^MLCT states in iron­(II) complexes by modulating ligand field strength and MLCT state energies.^4,6,7,9^ Additionally, efficient intersystem crossing (ISC) is required after initial population of the Franck–Condon (FC) state, which is driven by strong spin–orbit coupling (SOC).^18–20^ SOC can be invoked directly by orbital symmetry considerations as described by the El-Sayed rules,^21,22^ generalized by Marian,^18^ and by a heavy atom effect (that scales with *Z*_eff_^4^ for hydrogen-like atoms)^23^ or indirectly by vibronic SOC and spin-vibronic SOC.^18–20^ Besides earth-abundant TMCs, main group complexes of the abundant heavy elements might offer an alternative as photoluminescent compounds by contributing to the required SOC with the heavy atom effect.

Heavy main group complexes showing phosphorescence from triplet states and/or TADF from singlet states include mainly group 14 and 15 elements.^24–27^ For example, bismuth­(III) complexes with 6s^2^ electron configuration can show phosphorescence.^28–38^ Photoluminescent, isoelectronic group 14 complexes in the oxidation state + II with ns^2^ electron configuration, are very rare. The S_0_ ground state of heavy tetrylenes is characterized by an occupied and an unoccupied tetrel-centered orbital of high s and p character, respectively (Figure 1).^39–44^ Several types of ES are conceivable. Early studies by Vogler and co-workers on anionic halido tetrel complexes, tetranides [EX_3_]^−^ **I** ^**E,X**^ (E = Ge–Pb: X = Cl; E = Sn, Pb: X = Br) and [PbX_4_]^2–^ **II** ^**X**^ (X = Cl, Br) revealed phosphorescence from ^3^MC states at 510–604 nm after UV light excitation (Chart 1a).^45,46^ This ^3^MC state with s^1^p^1^ electron configuration is associated with a significant ES distortion from *C*_3v_ to *D*_3h_ symmetry in [EX_3_]^−^ complexes.^46^ This distortion fosters efficient nonradiative deactivation of the ES. Scandola and co-workers reported a weak green fluorescence and an even weaker red phosphorescence from intraligand charge transfer (ILCT) states for Pb­(QO)_2_ **III** (QOH = 8-hydroxyquinoline) in fluid and glassy solutions (Chart 1b).^47^ Bis­(β-diketonato)­lead­(II) complexes **IV** ^**R,R’**^ reported by Vogler and co-workers exhibit blue to green ILCT phosphorescence in the solid state.

**Figure 1:**
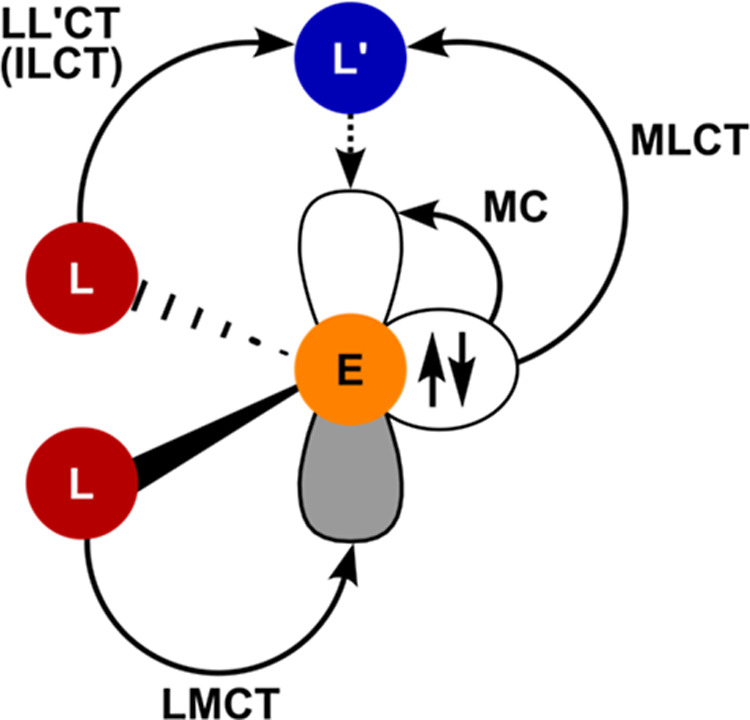
Schematic illustration of selected electronic transitions (excitations) in donor (L′) stabilized homoleptic tetrylenes EL_2_ (E = Si, Ge, Sn, Pb), IL and other possible LMCT (L′ → E) and MLCT (E → L) transitions omitted. Lone pair and p-orbital of E indicated.

**Chart 1:**
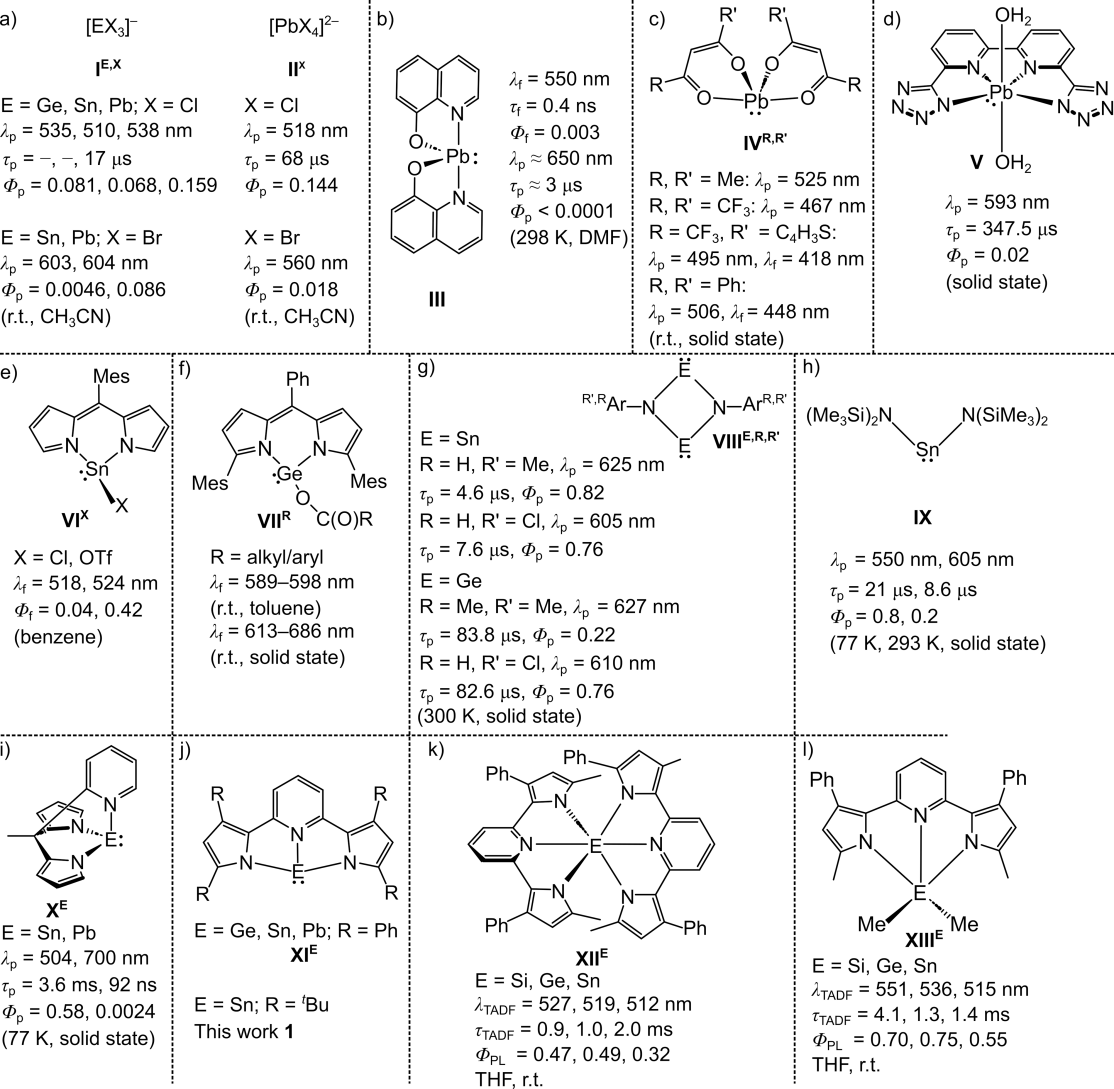
Tetrylene and tetrane complexes with selected photophysical data.

The phosphorescence intensity is reduced in solution at room temperature. The weak phosphorescence of these bis­(β-diketonato) plumbylenes was attributed to large amplitude motions in the ES leading to fast nonradiative relaxation.^48^ The respective hexafluoroacetylacetonato tin­(II) complex is nonemissive, even in the solid state.^48,49^ A series of lead­(II) coordination polymers with halido and polydentate tetrazolato ligands are phosphorescent from ^3^IL, ^3^MC and ^3^MLCT states with contribution of ^3^XLCT state character (X = halide). The phosphorescence lifetimes reach τ_p_ = 1.55 ms with a maximum quantum yield of Φ_p_ = 0.165 in the solid state. The monomolecular congener **V** with 6,6′-bis­(1*H*-tetrazol-5-yl)-2,2′-bipyridine as proligand shows ^3^MLCT phosphorescence with significantly lower phosphorescence quantum yield in the solid state as compared to the polymers (Φ_p_ = 0.02; Chart 1d).^50^ Substitution of a chlorido ligand with a weakly coordinating triflato (OTf^–^) ligand tremendously increases the fluorescence quantum yield of a dipyrrinato tin­(II) complex from 0.04 (**VI** ^**Cl**^) to 0.42 (**VI** ^**OTf**^) and bathochromically shifts the fluorescence band maximum from λ_f_ = 518 to 524 nm (Chart 1e).^51^ A series of dipyrrinato carboxylato germylenes **VII** ^**R**^ fluoresce at λ_f_ = 589–598 and at λ_f_ = 613–686 nm in toluene and in the solid state at room temperature, respectively (Chart 1f).^52^ Dodonov and co-workers described low-valent tin­(II) and germanium­(II) imides possessing a planar E_2_N_2_ core. [^H,Me^ArNSn]_2_ (^H,Me^ArNH = 2,6-Bis­[di­(phenyl)­methyl]-4-methylaniline) **VIII** ^**Sn,H,Me**^, for example, shows phosphorescence from a mixed ^3^[MC/LMCT] state at λ_p_ = 625 nm with τ_p_ = 4.6 μs and a high phosphorescence quantum yield of Φ_p_ = 0.82 in the solid state at 300 K. The emission is strongly diminished in solution (Φ_p_ = 0.003) (Chart 1g),^53^ suggesting that nonradiative processes are significantly accelerated in solution. The germanium congener [^H,Cl^ArNGe]_2_ **VIII** ^**Ge,H,Cl**^ reaches a high phosphorescence quantum yield of Φ_p_ = 0.76 in the solid state.^54^

Lappert’s diamino stannylene Sn­[N­(SiMe_3_)_2_]_2_ **IX** ^55,56^ shows thermochromic green to orange phosphorescence and thermally activated delayed phosphorescence (TADP) between 77 and 293 K arising from mixed ^3^[MC/LMCT] states (λ_p_ = 550 nm, τ_p_ = 21 μs, Φ_p_ = 0.8 in the solid state at 77 K, Chart 1h).^57^ The intramolecular donor-stabilized stannylene Sn­(bpep) **X** ^**Sn**^ (H_2_bpep = 2-[1,1-bis­(1*H*-pyrrol-2-yl)­ethyl]­pyridine) surpasses the lead congener Pb­(bpep) **X** ^**Pb**^ in terms of photophysical performance. **X** ^**Sn**^ shows green non-Kasha phosphorescence from a pyrrolide-to-pyridine ^3^ILCT state at λ_p_ = 504 and 523 nm at 77 K in the solid state and in solution, respectively (Chart 1i, Figure 2a).^58^ The emission lifetimes and photoluminescence quantum yields are strongly temperature dependent between 77 and 293 K both in solution (τ_p,av_ = 0.975 μs/<8 ns) and in the solid state (τ_p_ = 3.6 ms/14 ns, Φ_p_ = 0.58/0.005). This indicates that thermally activated nonradiative processes become dominant at ambient temperature. These were attributed to pyridine (or pyrrolide) donor dissociation with concomitant ^3^ILCT → ^3^LMCT internal conversion (IC) and fast nonradiative ISC from the ^3^LMCT state to GS.^58^ Sn­[N­(SiMe_3_)_2_]_2_ and Sn­(bpep) emit with similar emission wavelengths, yet from excited states with different character, namely ^3^[MC/LMCT] and ^3^ILCT, respectively. Pyridine donor coordination shifts the ^3^LMCT state of Sn­(bpep) to higher energy at the FC geometry and increases the formerly nonbonding p-type tetrel orbital in energy due to Sn–N^py^ σ antibonding character. Large amplitude motion, corresponding to pyridine donor dissociation, lowers the energy of this ^3^ILCT state significantly, enabling fast nonradiative decay via this mode.

**Figure 2:**
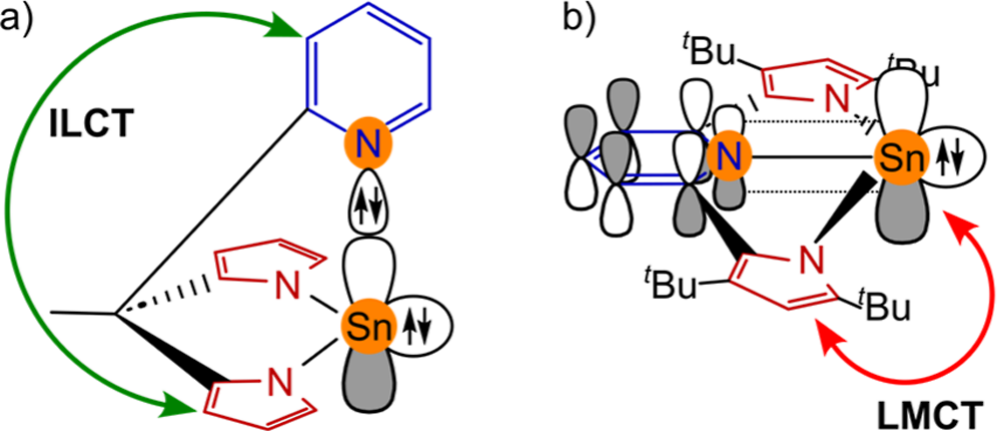
Conceptual approach to tune the character of the lowest-energy excited state of dipyrrolide/pyridine stannylenes by (a) *facial* and (b) *meridional* ligand topology.

The discussed tetrel­(II) compounds clearly indicate that a diverse manifold of ESs can be considered emissive, such as ILCT, MC, MLCT, LMCT or XLCT states and combinations thereof. This is schematically depicted for selected transitions in Figure 1. Furthermore, fluorescence or TADF from singlet states or phosphorescence from triplet states are possible radiative pathways, depending on the excited state energies and ISC efficiencies. Design concepts in combination with structure property relationships, as discussed in the context of photoactive TMCs, to selectively modify the nature and energy of ESs are not well established for tetrel­(II) compounds. Yet, a deeper understanding might give a rationale to design photoactive/photoluminescent tetrel­(II) compounds.

Eliminating the tin p-type orbital donor interaction in Sn­(bpep) can in principle prevent the pathway of nonradiative deactivation involving the EL′ mode. This consideration led us to investigate a ligand system analogous to bpep^2–^ with similar pyrrolide/pyridine donor properties, yet with different coordination topology by changing the tripodal bpep^2–^ with the meridionally coordinating pyridine dipyrrolide (pdp^2–^) pincer-type ligand [^*t*−Bu^pdp^*t*–Bu^]^2–^ (H_2_^*t*–Bu^pdp^*t*–Bu^ = 2,6-bis­(3,5-di-*tert*-butylpyrrol-2-yl)­pyridine, Chart 1j).^59^ In the latter meridional coordination, the pyridine donor should switch from a σ to a π donor interaction with the vacant tin p-type orbital (Figure 2). Switching the coordination topology from *facial* to *meridional* should lower the ^3^LMCT state, but leave the ^3^ILCT state energy essentially unaffected. This ligand coordination mode should furthermore inhibit pyridine donor dissociation as nonradiative deactivation mode. The ground state reactivity of the tetrylene complexes E­(^Ph^pdp^Ph^) **XI** ^**E**^ (E = Ge, Sn, Pb, H_2_^Ph^pdp^Ph^ = 2,6-bis­(3,5-diphenyl-1*H*-pyrrol-2-yl)­pyridine) with a related ligand system has been studied previously (Chart 1j).^60,61^ However, the photophysics of these systems remained unexplored. In contrast, the photophysical properties of tetrane­(IV) complexes with pdp-type ligands E­(^Me^pdp^Ph^)_2_ **XII** ^**E**^ and E­(Me)_2_(^Me^pdp^Ph^) **XIII** ^**E**^ (H_2_^Me^pdp^Ph^ = 2,6-bis­(5-methyl-3-phenyl-1*H*-pyrrol-2-yl)­pyridine) have been reported. These tetranes show prompt fluorescence and TADF with emission lifetimes in the millisecond range for the TADF process and high photoluminescence quantum yields (Chart 1k,l).^62,63^ Additionally, H_2_^Mes^pdp^Ph^, [H_3_^Mes^pdp^Ph^]Cl and Li_2_^Mes^pdp^Ph^ show intense fluorescence.^64^

This study sheds more light on the fundamental photophysical processes of tin­(II) complexes and connects topological ligand design concepts with experimental photophysical results. We discuss the synthesis and structure as well as emission properties of the Sn­(^*t*−Bu^pdp^*t*–Bu^) complex **1** (Chart 1j). The elucidation of these properties is supported by variable-temperature single crystal XRD analysis, variable-temperature steady-state and time-resolved emission spectroscopy as well as quantum chemistry calculations on the spin–orbit coupling (SOC) time-dependent (TD) density functional theory (DFT) level of theory.

## Results and Discussion

### Synthesis and Characterization of **1**

The tetrylene complex Sn­(^*t*−Bu^pdp^*t*–Bu^) **1** is synthesized by transamination of H_2_^*t*–Bu^pdp^*t*–Bu^ ^59^ with Sn­[N­(SiMe_3_)_2_]_2_ ^55,56^ in a 3:1 mixture of acetonitrile and diethyl ether as an orange red, extremely moisture- and air-sensitive solid in 44% yield (Scheme 1).

**Scheme 1:**
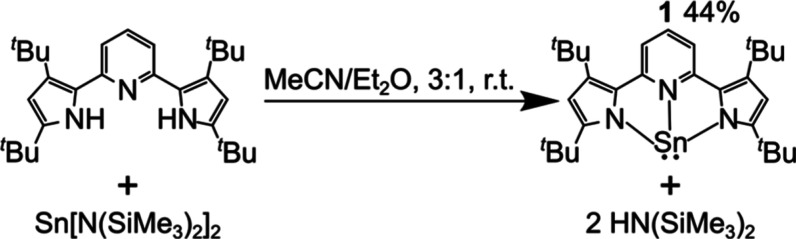
Synthesis of Stannylene Sn­(^*t*−Bu^pdp^*t*–Bu^) **1** via Transamination from Sn­[N­(SiMe_3_)_2_]_2_ and H_2_^*t*–Bu^pdp^*t*–Bu^

**1** is well soluble in tetrahydrofuran (THF) but barely soluble in *n*-pentane or benzene despite the four *tert*-butyl substituents. The coordinating nature of THF might be responsible for the enhanced solubility in THF, most likely forming a thf adduct. THF coordination has been observed in a previous study with the related complex **XI** ^**E**^ forming the bis­(thf) adduct XI^E^(thf)_2_.^60^ Donor-free **1** has been fully characterized by elemental analysis and APCI^+^ mass spectrometry. **1** has been investigated as donor-free and as thf adduct by solid state (^119^Sn) and solution (^1^H, ^13^C, ^119^Sn) NMR spectroscopy, respectively (Figures S1–S9). The ^119^Sn NMR resonance of solid **1** appears at δ = −101.6 ppm (Figure S9). The ^119^Sn NMR resonance of **1**(thf-*d*_8_)_2_ in THF-*d*_8_ appears with δ = −466.5 ppm in the chemical shift range of analogous complexes (Figure S8),^60^ indicative for thf adduct formation (Scheme 2a), analogous to the behavior of Sn­[N­(SiMe_3_)_2_]_2_.^65^ The adduct formation leads to a formal occupation of the p-type tetrel orbital with concomitant decreased paramagnetic (de)­shielding.^66^

**Scheme 2:**
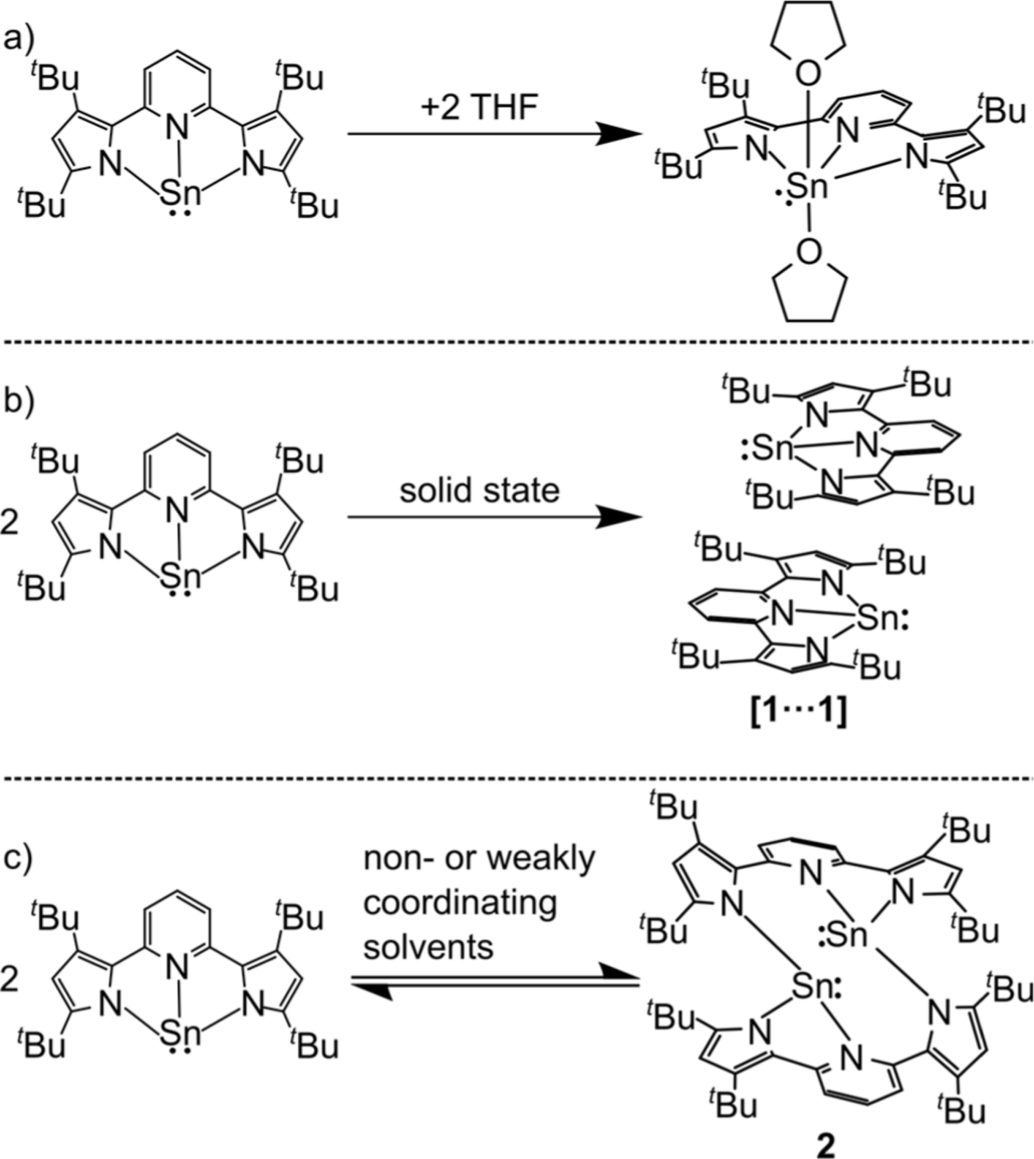
Formation of (a) a thf Adduct, (b) a Van-der-Waals Dimer **[1···1]** in the Solid State, and (c) a Bridged Dimer **2** in Non- or Weakly Coordinating Solvents from Sn­(^*t*−Bu^pdp^*t*–Bu^) **1**

The absence of coordinating donors in the solid state is confirmed by single crystal X-ray diffraction (SC-XRD) analysis. Crystals of **1**, suitable for SC-XRD analyses, were obtained from a saturated acetonitrile solution by slow evaporation of the solvent. **1** crystallizes in the triclinic space group P1̅ with one molecule in the asymmetric unit (Figure 3a, Table S1). For the understanding of the photophysical properties (vide infra), a SC-XRD analysis was performed at variable temperatures, which ruled out a phase transition (100–225 K, Table S2). The stannylene features a T-type coordination of the nitrogen donors with a pyramidalized coordination of the Sn atom with a nitrogen­(pyrrolide1)–tin–nitrogen­(pyrrolide2) (N^pyr1^–Sn–N^pyr2^) angle of 117.07(6)° and an angle sum (∑(N–Sn–N)) of 262.16°. The tin–nitrogen bond lengths of the pyridine (Sn–N^py^) and pyrrolide donors (Sn–N^pyr1^/Sn–N^pyr2^) amount to 2.168(2) and 2.220(2)/2.197(2) Å at 120 K, respectively (Table S1). These bond parameters remain constant within the 3σ limit in the 100–225 K temperature range (Table S1). Two molecules **1** form a centrosymmetric pair **[1···1]** with a slipped head-to-tail arrangement (Figure 3b, Scheme 2b). These pairs stack along the *b* axis of the unit cell (Figures S10–S12). In the **[1···1]** pair, the tin atoms have short intermolecular contacts to the pyridine carbon atoms (C17′) of the partner molecule (Figure 3b, S13). Interestingly, the Sn–C17′ distance within the **[1···1]** pair significantly increases from 3.694(2) to 3.747(2) Å between 100 and 225 K, but remains slightly below the sum of van-der-Waals radii of Sn and C (3.87 Å).^67^ We denote this **[1···1]** pair a van-der-Waals dimer. The DFT calculations to **1** and **[1···1]** reproduce the experimental structural parameters obtained from SC-XRD analyses, including the pyramidalization of the tin atom (N^pyr1^–Sn–N^pyr2^: 117.04°, **1**) and the Sn–C17′/Sn′–C17 contacts (3.637/3.638 Å, **[1···1]**) (Figure 3c, S13, Table S1). The formation of geometry relaxed **[1···1]** is only slightly preferred to **1** by Δ*G* = −8 kJ mol^–1^, according to DFT calculations (CPCM­(hexane)-RIJCOSX-B3LYP-D3BJ-ZORA-SARC/J-ZORA-def2-TZVPP/SARC-ZORA-TZVPP­(Sn)). A quantum theory of atoms in molecules (QTAIM) analysis reveals bond critical points for the Sn–C17′/Sn′–C17 contacts (Figure S13c), indicating a weak noncovalent interaction, according to the electron density ρ = 0.00711 au (≤0.03 au), Laplacian of electron density ∇^2^ρ = 0.0164 au (>0 au), energy density *H*(***r***) = 0.000600 au (>0 au), and a ratio between Lagrangian kinetic energy *G*(***r***) and potential energy density *V*(***r***) of −*G*(***r***)/*V*(***r***) = 1.21 (>1).^68^

**Figure 3:**
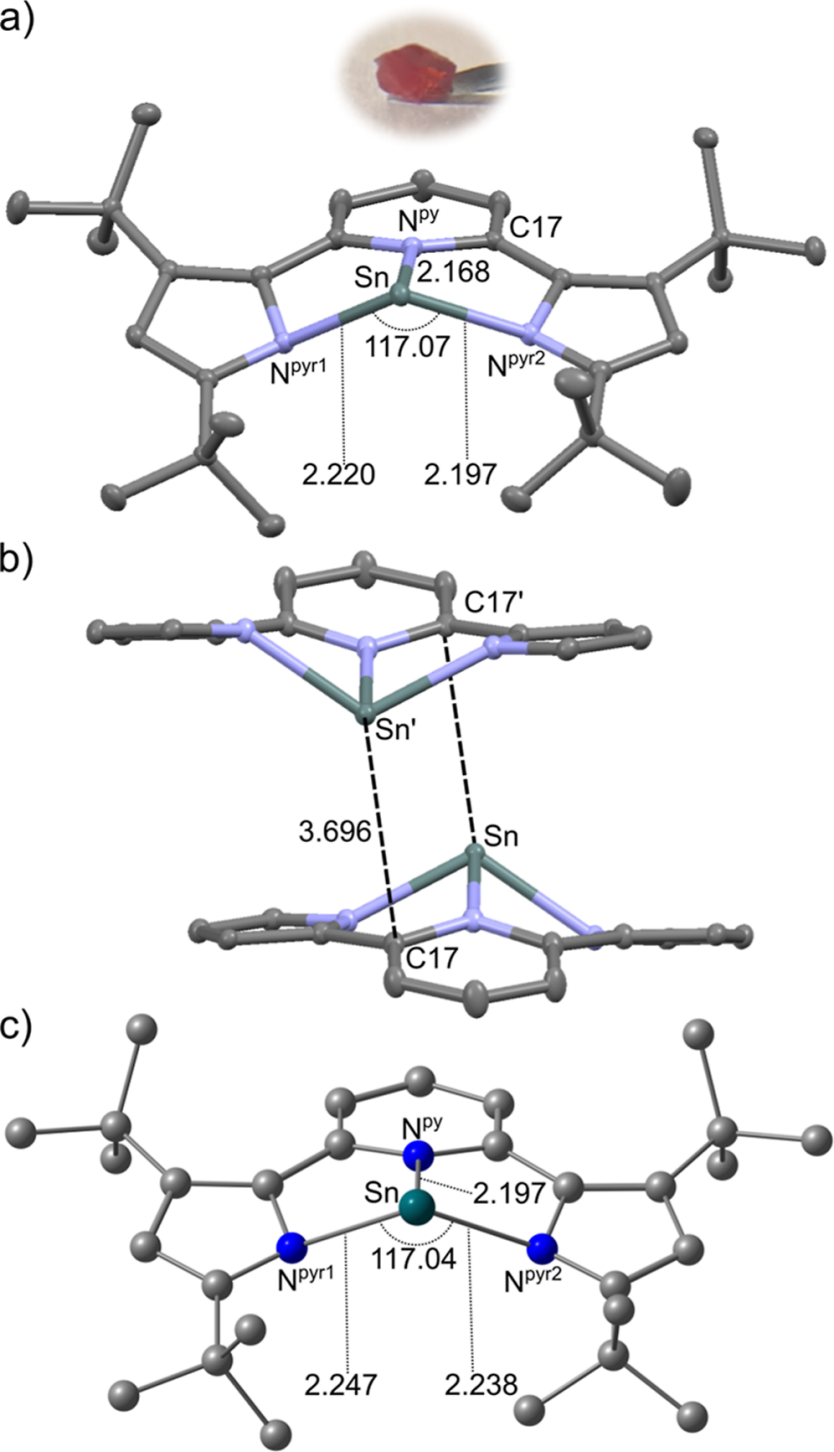
(a) Molecular structure of **1** with selected structural parameters in Å and degrees [°] from SC-XRD analysis (photograph of a red crystal of **1** obtained by slow evaporation of an acetonitrile solution of **1**). (b) Centrosymmetric pairs **[1···1]** with intermolecular Sn···C17′ contacts at 120 K (thermal ellipsoids at 50% probability level). (c) Molecular structure of **1** from DFT calculations (CPCM­(hexane)-RIJCOSX-B3LYP-D3BJ-ZORA-SARC/J-ZORA-def2-TZVPP/SARC-ZORA-TZVPP­(Sn)). Hydrogen atoms omitted.

In non- or weakly coordinating solvents like benzene, **1** slowly dimerizes to **2** with bridging pdp^2–^ ligands, as already observed for the phenyl-substituted derivative **XI** ^**E**^ (Scheme 2c).^60^ The slow dimerization can be followed by ^1^H NMR and DOSY ^1^H NMR spectroscopy (Figures S14–S19) showing a double set of ^1^H NMR resonances for the ligand protons and a separate trace with a smaller diffusion coefficient for **2** in the DOSY spectra. A ratio for **1**/**2** of ≈1:0.125 and ≈1:0.42, the latter representing the equilibrium ratio, is established in benzene 40 min and 6 h after sample preparation of a 48 mM solution of **1**, respectively, as estimated by integration of the *tert*-butyl ^1^H NMR resonances (Figure S14–S17). Addition of THF-*d*_8_ to the equilibrated mixture of **1**/**2** furnishes the thf adduct of **1**, likely 1­(thf-*d*_8_)_2_ (Figure S15b). An ≈1:0.40 ratio of **1**/**2** is already obtained in 3-methylpentane 30 min after sample preparation, indicative for a faster monomer/bridged dimer equilibration (Figure S19). The diffusion coefficients from DOSY NMR spectroscopy amount to 7.09 × 10^–10^ m^2^ s^–1^ and 5.71 × 10^–10^ m^2^ s^–1^ for **1** and **2**, respectively (Figure S18). The derived hydrodynamic radii of 4.7 and 5.9 Å (1:0.80 ratio) fit well to the estimated SC-XRD molecular radii of 5.4 and 7.1 Å (1:0.76 ratio) of **1** and **2**, respectively. As confirmed by SC-XRD analysis, the structure of the bridged dimer **2** is similar to that of reported [XI^E^]_2_ with μ-κ^1^N^pyr1^:κ^2^N^pyr2^, N^py^ [^R1^pdp^R2^]^2–^ bridging ligands (Figure S20).^60^ The bridged dimer **2** is stabilized by Δ*G* = −66 kJ mol^–1^ to donor-free **1**, according to DFT calculations.

The UV/vis absorption spectrum of a mixture of **1** with the bridged dimer **2** in *n*-pentane is a superposition of the TDDFT calculated spectra of **1** and **2** shifted by +3500 cm^–1^ and −1650 cm^–1^, respectively (Figure S21a,c), further confirming the dimerization in noncoordinating solvents. The spectrum at 77 K, 25 min after sample preparation, yields essentially the same pattern as at 293 K with a more structured low-energy band due to band narrowing at low temperatures (Figure S21d). This low-energy band could be fitted with three Voigt functions supporting the TDDFT calculations of **1** and **2** (Figure S21e).

The local coordination geometry of the tin ions in **2** resembles that of Sn­(bpep) with σ-coordination of a pyridine to the p­(Sn) orbital. According to the difference electron densities and charge transfer number analysis, the lowest energy electronic transition of **2** at around 460 nm (shifted by −1650 cm^–1^) is of predominant ILCT character fully analogous to Sn­(bpep) (Figures S22 and S23).^58^ In addition, the emission properties of **2** with its emission band peaking at λ_p_ = 540 at 293 K in solution are very similar to that of Sn­(bpep) with λ_p_ = 535 nm at 293 K in the solid state (Figure S24).^58^ Due to the thf adduct formation in THF and the dimerization in aromatic and aliphatic solvents, which result in p-type orbital occupation and hence to different excited state energies, a detailed comparative study of monomeric *meridional* Sn­(^*t*−Bu^pdp^*t*–Bu^) **1** and the monomeric *facial* stannylene Sn­(bpep) (Figure 2) is not feasible in solution. Consequently, we focused on the investigation of solid **1** without any additional donors, i.e. coordinating solvent, and without dimerization to **2**.

### Optical Properties and Excited State Dynamics of Solid **1**

DFT calculations on **1** (Figures 4 and S25) confirm the frontier molecular orbital order as envisaged in Figure 2. The molecular orbital of high Sn p character in **1** is significantly stabilized by 0.75 eV (LUMO) through π conjugation with the pyridine, compared to Sn­(bpep), with the p­(Sn) orbital lying in the pyridine plane and interacting with the pyridine lone pair. The ligand-centered unoccupied molecular orbital of high pyridine character (LUMO+2) is destabilized by 0.80 eV (Figures 4 and S25). These effects invert the LUMO characters as predicted by the qualitative model (LUMO inversion). The meridional coordination mode of [^*t*−Bu^pdp^*t*–Bu^]^2–^ allows pyrrolide/pyridine π orbital conjugation resulting in HOMO–1, HOMO and LUMO+2 of mixed pyrrolide/pyridine character, as compared to related MOs in Sn­(bpep) (Figure 4). Furthermore, the meridional coordination mode enables pyrrolide and pyridine π orbital admixtures to the tin s- and p-type orbitals, respectively.

**Figure 4:**
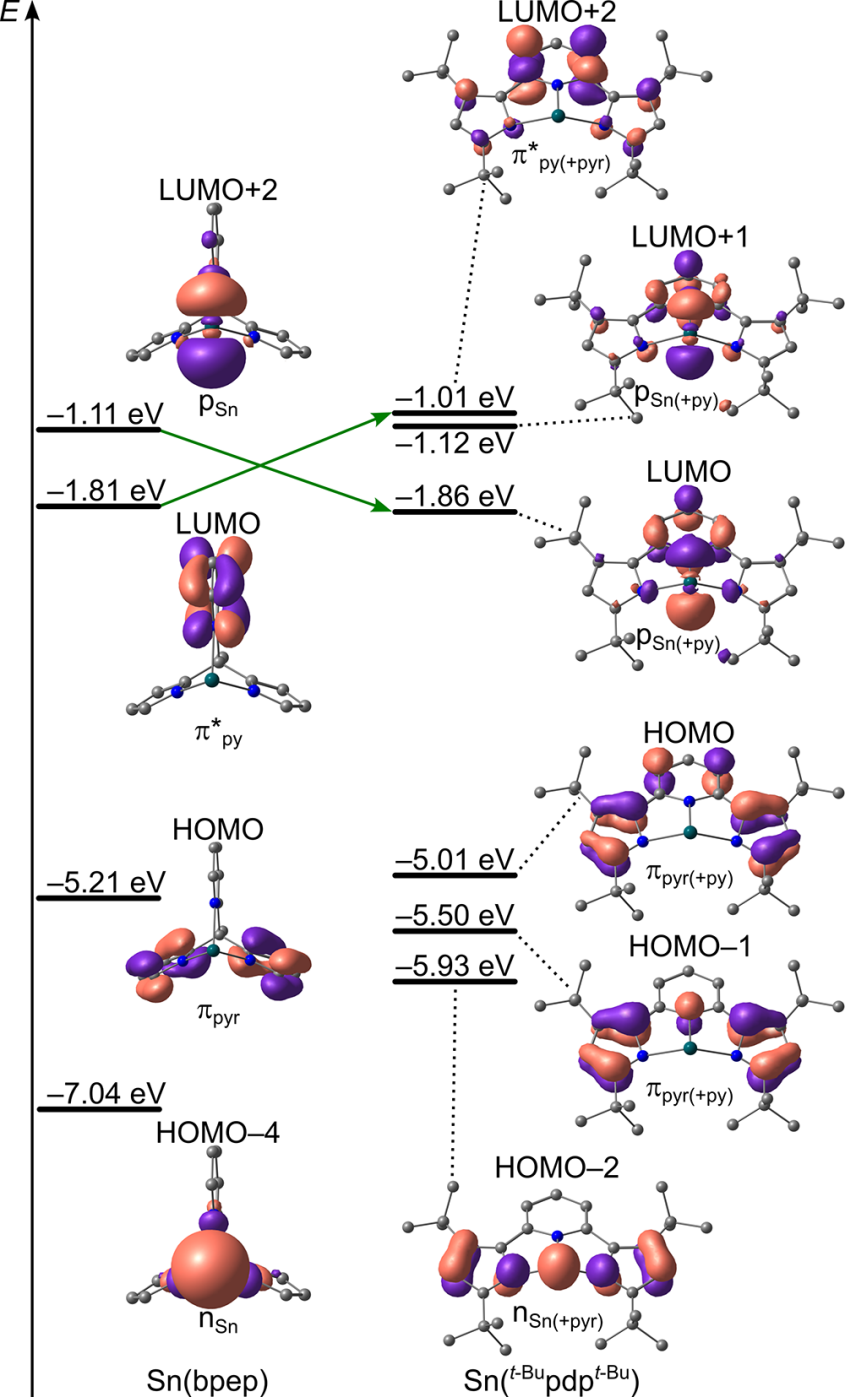
Calculated frontier orbitals with energies of Sn­(bpep)^58^ and Sn­(^*t*−Bu^pdp^*t*–Bu^) classified according to the dominant orbital character illustrating the LUMO inversion (isosurface value: 0.05 au). CPCM­(hexane)-RIJCOSX-B3LYP-D3BJ-ZORA-SARC/J-ZORA-def2-TZVPP/SARC-ZORA-TZVPP­(Sn).

TDDFT calculations of geometry-optimized **1** deliver a spin-allowed transition of high ^1^LMCT character in the visible spectral region (all transitions shifted by +3500 cm^–1^), namely an intense S_0_ → S_1_ (HOMO→LUMO) transition at 426 nm (blue), responsible for the orange red color. Weak S_0_ → S_2_ (HOMO–1→LUMO) and intense S_0_ → S_3_ (HOMO→LUMO+1) transitions at 335 nm and 371 nm, respectively, are of high ^1^LMCT character as well (Figures 5, S26 and S27). The lowest-energy transition of dominant ^1^ILCT (pyrrolide → pyridine) character is found at 303 nm (S_0_ → S_5_, HOMO–1→LUMO+1 (35%), HOMO–1→LUMO+2 (53%), S26 and S27). In contrast, the lowest-energy ^1^ILCT transition (HOMO–LUMO) of Sn­(bpep) is calculated at lower energy (412 nm).

**Figure 5:**
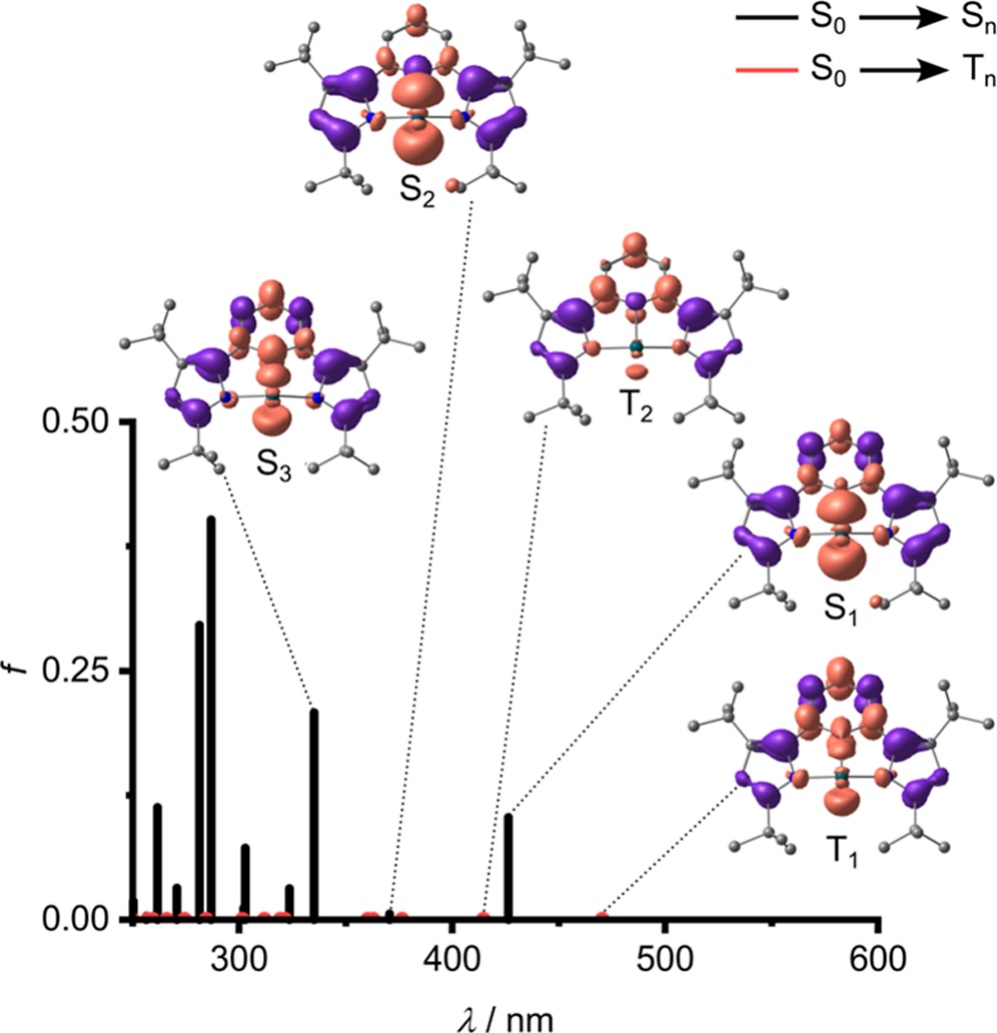
TDDFT calculated oscillator strengths of **1** for S_0_ → S_*n*_ transitions, position of S_0_ → T_*n*_ transitions marked by red points with difference electron densities of selected characteristic transitions (isosurface value 0.003 au, purple = electron loss, orange = electron gain). CPCM­(hexane)-RIJCOSX-B3LYP-D3BJ-ZORA-SARC/J-ZORA-def2-TZVPP/SARC-ZORA-TZVPP­(Sn).

Spin-forbidden singlet–triplet transitions S_0_ → T_n_ of varying LMCT/ILCT character are calculated in the visible spectral region (Figure 5). The S_0_ → T_1_ and S_0_ → T_2_ transitions correspond to changes in the HOMO → LUMO and HOMO–1 → LUMO (60%)/HOMO → LUMO+2 (32%) occupations, respectively. At the FC geometry, the T_1_ and T_2_ states are of mainly ^3^LMCT and ^3^ILCT character with some admixed ^3^LMCT character, respectively. While the T_1_ FC state is lower in energy than the S_1_ state by 0.27 eV, the T_2_ FC state is almost degenerate with the S_1_ FC state. Unfortunately, recording solid-state absorption spectra for comparison with the calculated transitions was prohibited by the extreme air- and moisture-sensitivity of **1**. However, the UV/vis absorption spectrum of **1** (+**2**) does show a maximum around 426 nm which (in part) corresponds to the S_0_ → S_1_ transition of **1** (Figure S21).

Excitation at λ_exc_ = 450 nm of **1** in the solid state results in a red emission peaking at 710 nm (Figure 6a). The emission lifetime amounts to τ_p,293_ = 221 ns with a photoluminescence quantum yield of Φ_p,293_ = 0.0044 at 293 K, respectively (Figure S28, Table S3). The long lifetime suggests phosphorescence as emissive process. Upon cooling to 77 K, the maximum shifts hypsochromically to 690 nm by 408 cm^–1^ (Figures 6a and S29). Compared to Sn­(bpep) with λ_p,77_ = 504 nm, the emission of **1** at λ_p,77_ = 690 nm is significantly red-shifted by 5350 cm^–1^ as expected from the frontier molecular orbital energies (Figure 4).^58^ At 77 K, lifetime and quantum yield increase to τ_p,77_ = 128 μs and Φ_p,77_ = 0.0116, respectively (Figure S30, Table S3). The emission lifetime at 77 K τ_p,77_ was fitted biexponentially, yet the second fraction amounts to only 3% (τ_p2,77_ = 313 μs), which can be caused by minor defects on the surface of the solid and is hence neglected in the following discussion (Figure S30). Excitation spectra (λ_obs_ = 700 nm), recorded between 293 and 77 K, are essentially superimposable (Figure S31). According to the TDDFT calculations, the absorption maximum at λ_max_ = 426 nm of the absorption spectrum at 77 K of a mixture of **1** and **2** in *n*-pentane is dominated by the S_0_ → S_1_ absorption of monomeric **1** and this is reflected in the excitation spectrum of solid **1** with λ_max_ = 420 nm (Figures S20 and S31). As absorption and filter effects distort the excitation spectra of solid **1** at higher energy, the excitation spectra diverge from the absorption spectra at λ < 400 nm.

**Figure 6:**
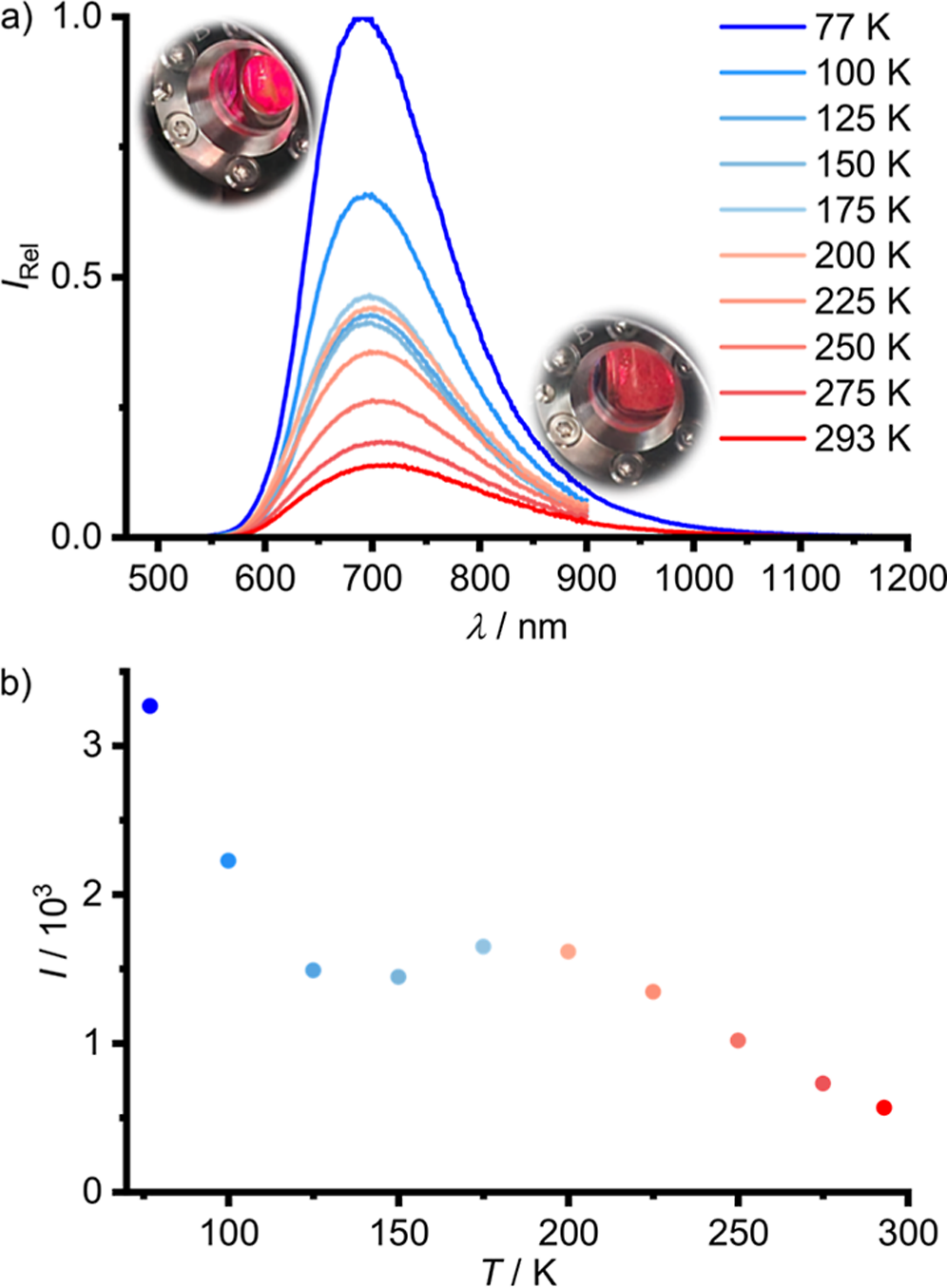
(a) Temperature-dependent phosphorescence spectra of **1** in the solid state between 77 K (blue) and 293 K (red) with λ_exc_ = 450 nm. (Inset: Photographs of the red photoluminescence at 77 K, left and 293 K, right). (b) Integrated phosphorescence emission intensity of **1** in the solid state between 77 and 293 K with λ_exc_ = 450 nm.

Assuming a unity ISC quantum yield, the nonradiative and radiative rate constants *k*_nr_ and *k*_r_ were determined from the phosphorescence lifetimes and quantum yields at 77 and 293 K as *k*_nr,77_ = 7700 s^–1^, *k*_nr,293_ = 4.5 × 10^6^ s^–1^ and *k*_r,77_ = 100 s^–1^, *k*_r,293_ = 20,000 s^–1^. While the increased nonradiative decay at higher temperature is expected, the faster radiative decay at higher temperature suggests a thermally populated higher energy state with a higher radiative rate constant.

Detailed variable-temperature studies reveal that the integrated emission intensity maximizes at 175 K and decreases above and below 175 K (Figure 6b). Below 125 K, the integrated emission intensity increases again which is expected behavior (Figure 6b). The peculiar effect with a maximum intensity is reproducible both for cooling and heating temperature ramps. Additionally, it is fully reversible persisting for several cooling/heating cycles (Figures S32 and S33) and is independent from excitation wavelength (λ_exc_ = 420, 450, 480 nm), ruling out a temperature-dependent shift or narrowing of the absorption bands as underlying effects (Figures 6, S34, and S35).

The phosphorescence rate constants *k*_obs_(*T*) = 1/τ_p_(*T*) were fitted with two independent thermally activated processes (*E*_a,1_; *E*_a,2_; *A*_1_; *A*_2_) according to an Arrhenius model (eq 1, Figure 7a, Table S4). The first process is essentially temperature-independent with a negligible activation barrier of *E*_a,1_ = 0.01 eV and a small preexponential factor *A*_1_ = 4.40 × 10^4^ s^–1^, while the second process has a barrier of *E*_a,2_ = 0.19 eV with *A*_2_ = 7.86 × 10^9^ s^–1^. To further elucidate the temperature dependence of the radiative and nonradiative processes with rate constants *k*_r_ and *k*_nr_ (*k*_obs_ = *k*_nr_ + *k*_r_), individual Arrhenius analyses were performed for *k*_r_ and *k*_nr_. To this end, the relative phosphorescence quantum yields were determined by integrating the emission bands over an energy scale from 11,111 to 18,900 cm^–1^ referenced to the phosphorescence quantum yield, which was determined absolutely at 293 K as Φ_p,293_ = 0.0044 (Table S5). The phosphorescence quantum yields at 77 K determined absolutely (Φ_p,77_ = 0.0116) and by the relative method (Φ_p,rel,77_ = 0.024) agree sufficiently well, considering the small absolute photoluminescence quantum yield, validating the procedure. The Arrhenius fit (eq 1) for *k*_nr_ gives the same activation barriers *E*_a,1_/*E*_a,2_ and similar preexponential factors *A*_1_/*A*_2_ as the fit for *k*_obs_ (Figures 7a and S36, Tables S3 and S4).

1 $$k_{j}=A_{i}\cdot e^{-E_{a,i}/k_{B}T}$$

with *j* = *r*, *nr*, *A*_*i*_ preexponential factor, *E*_*a*,*i*_ activation energy, *k*_B_ Boltzmann constant and *T* temperature.

**Figure 7:**
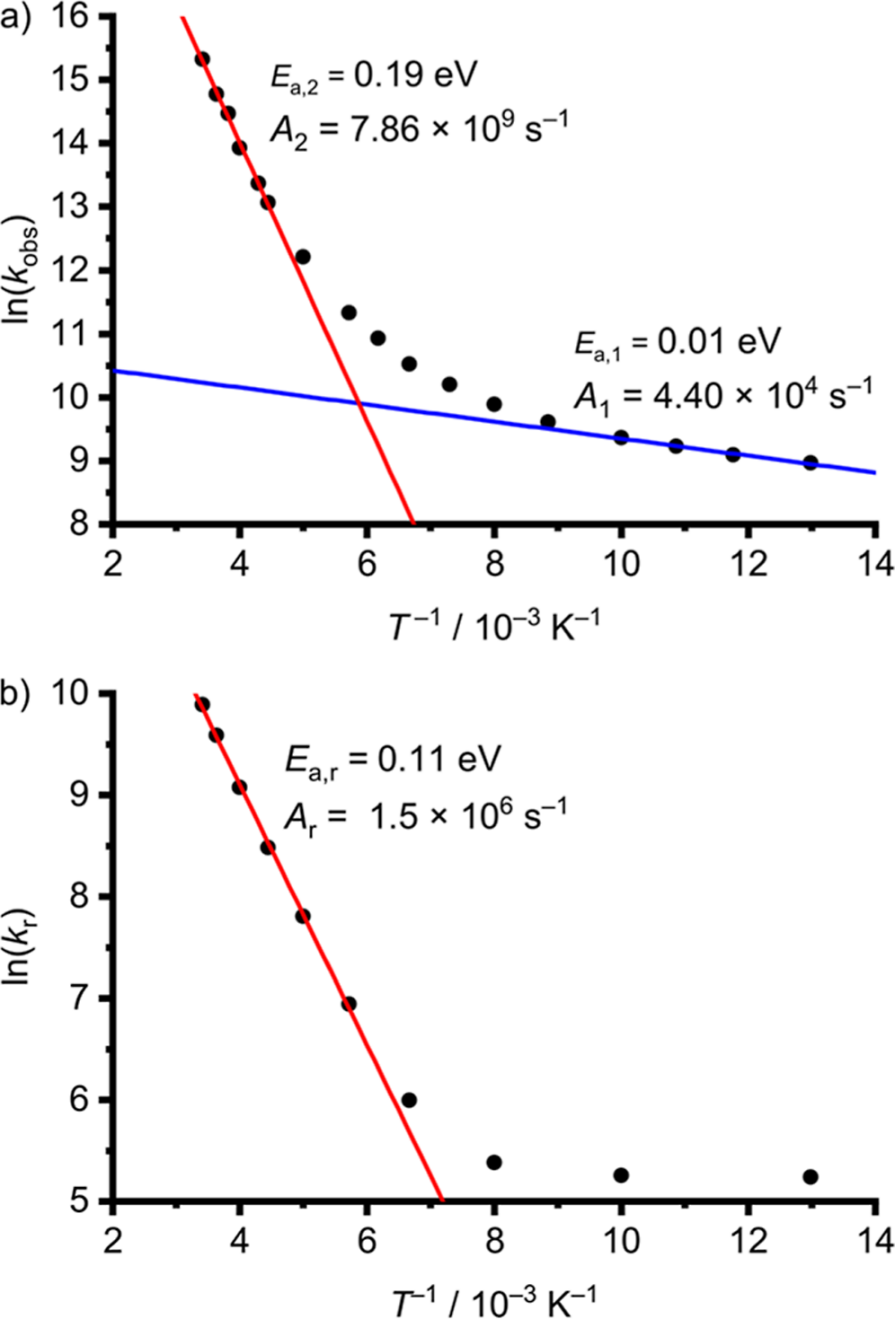
(a) Arrhenius plot of the observed photoluminescence rate constants (*k*_obs_(*T*) = 1/τ_p_(*T*), *T* = 77–293 K) with fits of the high and low temperature regimes in red and blue. (b) Arrhenius plot of the radiative rate constants *k*_r_(*T*), *T* = 77–293 K with fit of the high temperature regime in red.

This indicates that nonradiative processes dominate the excited state deactivation as already obvious from the low photoluminescence quantum yields. The energy barrier *E*_a,2_ = 0.19 eV might be tentatively ascribed to thermally activated T_1_ → S_0_ ISC. Unexpectedly, *k*_r_ also shows a strong temperature dependence with *k*_r,77_ = 100 s^–1^ and *k*_r,293_ = 20,000 s^–1^ at 77 and 293 K, respectively (Figure 7b, Tables S3 and S4). Between 77 and 125 K, *k*_r_ is essentially temperature independent. Above 175 K, a thermally activated radiative process with a barrier *E*_a,r_ = 0.11 eV and a preexponential factor *A*_r_ = 1.5 × 10^6^ s^–1^ becomes dominant (Figure 7b, Tables S3 and S4). In other words, a second emissive state at slightly higher energy, which has a significantly larger *k*_r_, is populated at higher temperatures.

The emission band energy and band shape do not change significantly with temperature (Figure 6), indicating that the two emissive states have a similar energy gap to the S_0_ state and a similar electronic character. Several models can be invoked to describe the thermal population of a slightly higher-energy emissive state, yet with an appreciable activation barrier of *E*_a,r_ = 0.11 eV. Four conceivable models A–D are depicted in Scheme 3. In model A, the higher emissive state is the T_2_ state, which is thermally populated by back-IC (TADP), while in model B, the higher emissive state is the S_1_ state, which is populated by back-ISC (TADF). Model C accounts for the splitting of the T_1_ triplet state into the M_S_ = 0, ±1 sublevels by SOC. This would be similar to the situation encountered by Ir­(ppy)_3_ with strong SOC enabled by Ir and a high-energy brighter M_S_ state with zero field splitting (ZFS) of 170 cm^–1^.^69,70^ Model D includes excitonic coupling of the van-der-Waals dimer **[1···1]** (Scheme 3b).

**Scheme 3:**
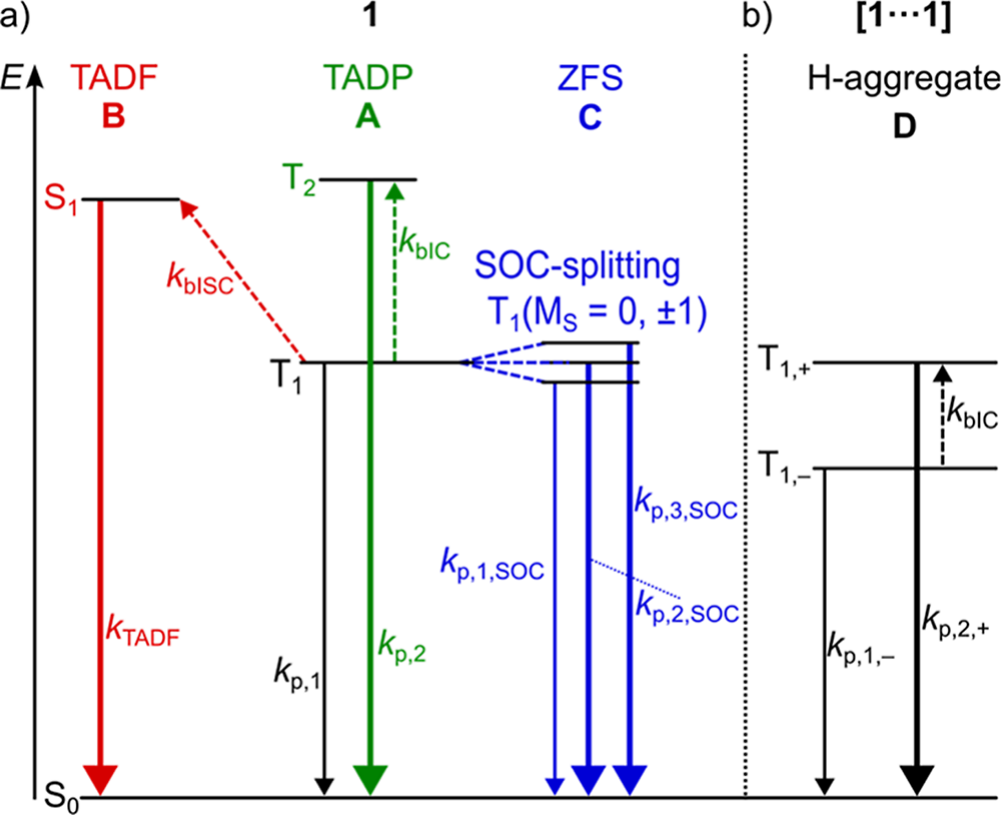
Conceivable Models for the Observed Thermally Activated Photoluminescene in **1**: (a) A: T_1_ → T_2_ Back-IC with T_2_ Phosphorescence (Green, TADP), B: T_1_ → S_1_ Back-ISC With fluorescence from S_1_ (Red, TADF), C: SOC-Split T_1_ State with Thermal Population of the M_S_ = 0, ±1 Sublevels with Different *k*_p_ (Blue, ZFS) and (b) D: Excitonic Coupling in the van-der-Waals Dimer **[1···1]**

To gain deeper insight into the nature of the emissive states and the pathway to these states, (SOC-TD)­DFT calculations were performed on S_0_, T_1_ and T_2_ states of monomeric **1** and on excited states of the van-der-Waals dimer **[1···1]** that is present in the solid state. We start the discussion with the excited state landscape of monomeric **1**. At the FC geometry, S_1_ and T_2_ states of **1** are nearly degenerate (Figure 5), which could allow for fast S_1_ → T_2_ ISC in the FC region after S_0_ → S_1_ excitation. Compared to the calculated SOC constant for the S_1_/T_1_ state pair SOCC­(S_1_/T_1_) = 31 cm^–1^, the corresponding constant for the S_1_/T_2_ state pair SOCC­(S_1_/T_2_) = 106 cm^–1^ is much larger. This can drive S_1_ → T_2_ ISC already in the FC region (Table S6). The ISC is then followed by T_2_ → T_1_ internal conversion (IC) and vibrational relaxation (VR). The relaxed geometries of the T_1_ and T_2_ states were calculated by DFT (excited state) optimizations. The relaxed T_1_ and T_2_ states are characterized by a planar and a pyramidal T-type coordination geometry with interplanar angles of 0° and 20° between the N^pyr1^/Sn/N^py^ and N^pyr1^/Sn/N^py^ planes, respectively (Figure S37). Furthermore, the N^pyr1^–Sn–N^pyr2^ angles slightly differ in the T_1_ and T_2_ states with 141° and 136°, respectively. This places the T_2_ state closer to the FC region, i.e. the S_0_ geometry (Table S1, Figure S37). The energies of the T_1_ and T_2_ states vary similarly along the N^pyr1^–Sn–N^pyr2^ bending mode with T_1_/T_2_ energy gaps of ≈ 0.3–0.5 eV according to a relaxed potential energy surface scan (Figure S38). These calculated T_1_/T_2_ energy gaps are too high to account for the experimentally observed thermally activated photoluminescence phenomenon with *E*_a,r_ = 0.11 eV (vide supra). With this argumentation, thermally activated T_1_ → T_2_ back-IC does not appear feasible, and the high-temperature emission does not occur from the T_2_ state (Scheme 3, model A). Additionally, the calculated T_1_/S_0_ gap of 0.86 eV at the relaxed flattened T_1_ state geometry is way too small compared to the experimentally determined emission energy of 1.80 eV. This finding indicates that geometry relaxation (flattening and N^pyr1^–Sn–N^pyr2^ bending) and the concomitant energy gain are hampered by the rigid environment of the solid. Similarly, the S_1_ state is higher in energy by ≈ 0.18–0.26 eV than the T_1_ state, so that TADF (Scheme 3, model B) appears unfeasible. Concerning model C, the splitting of the T_1_ state into its M_S_ = 0, ±1 sublevels by SOC has been calculated by SOC-DFT on the relaxed T_1_ geometry as 1.1 cm^–1^ (Scheme 3, model C). This appears to be too small to account for the much larger experimentally determined barrier. As models A–C fail to explain the experimental data (small energy gap allowing for thermal population, but medium large barrier between the two emissive states), model D—excitonic coupling—is considered. In the solid state, **1** forms van-der-Waals dimers **[1···1]** of slipped head-to-tail arranged molecules of **1** with intermolecular Sn···C contacts of around 3.7 Å (Figure S13, vide supra). In dimers or aggregates of dyes, the excitonic states can split into two levels through the interaction of electric transition dipoles and such an excitonic coupling may be present in **[1···1]**. In an H aggregate of **1**, the transition dipoles of the T_1_ state would add in the upper T_1,+_ state, but cancel in the lower T_1,–_ state (Scheme 3).^71–73^ Hence, the upper and lower T_1,+_ and T_1,–_ states would possess larger and smaller transition dipole moments, respectively. This would account for the experimentally observed high and low radiative rate constants at high and lower temperature of *k*_r,293_ = 20,000 cm^–1^ and *k*_r,77_ = 100 cm^–1^, respectively. In this model D, we equate the experimentally determined activation barrier Δ*E*_a,r_ = 0.11 eV of the radiative process (Figure 7b) to the barrier for T_1,–_ → T_1,+_ bIC, while the state splitting itself is comparably small. To support the proposed excitonic coupling, we performed SOC-TDDFT calculations on a truncated model van-der-Waals dimer **[1···1]** ^**H**^ based on the SC-XRD determined GS geometries (vide supra) with the *tert*-butyl substituents replaced by hydrogen atoms to reduce computational costs (Figure S39). TDDFT calculated transition densities of relevant S_0_ → S_1_, S_0_ → S_2_, S_0_ → T_1_ and S_0_ → T_2_ transitions of **1** lack densities on the *tert*-butyl substituents validating the truncated van-der-Waals dimer model (Figure S40). This van-der-Waals dimer model at the FC geometry obtained from SC-XRD delivers split levels for each monomer transition S_0_ → S_1_, S_0_ → T_1_ and S_0_ → T_2_ with averaged energy gaps of 430 cm^–1^, 22 cm^–1^ and 71 cm^–1^ for S_1,–_/S_1,+_, T_1,–_/T_1,+_ and T_2,–_/T_2,+_ states, respectively (Table S7). The calculated oscillator strengths averaged over the respective three M_S_ triplet sublevels amount to zero and 3.2 × 10^–6^ for the T_1,–_ and T_1,+_ states, respectively, supporting the proposed H aggregate excitonic coupling (Figure S41). The SOC-TDDFT calculated T_1,–_/T_1,+_ excitonic splitting of 0.003 eV (22 cm^–1^) for **[1···1]** ^**H**^ at the FC geometry is quite small accounting for the essentially constant emission band energy (Figure 6a). The validity of the results obtained from SOC-DFT calculations with the B3LYP functional has been tested by performing comparative calculations with the CAM-B3LYP, M06–2X, and TPPSh functionals on **[1···1]** ^**H**^ (@100 K). The latter two essentially support the findings from the calculations with the B3LYP functional (Table S8). The experimental barrier *E*_a,r_ = 0.11 eV pertains to the barrier for the T_1,–_/T_1,+_ IC process in this model D, which is not covered by the DFT calculations at the FC geometry.

The phosphorescence of **1** with a pincer-type ligand is significantly red-shifted compared to the phosphorescence of Sn­(bpep) with a tripodal ligand due to a change in electronic character from ^3^ILCT of Sn­(bpep) to ^3^LMCT of **1** (LUMO inversion). The peculiar temperature-dependence of the emission intensity of **1** in the solid state likely arises from excitonic coupling in an H aggregate **[1···1]** and thermally activated phosphorescence from the higher-lying brighter triplet excitonic state.

## Summary and Conclusion

Controlling the interaction of the tin­(II) p­(Sn) orbital in dipyrrolide stannylenes with a pyridine donor by the dipyrolato/pyridine ligand topology (tripod/pincer) switches the σ-donor­(pyridine) → p­(Sn) interaction in Sn­(bpep) to a π-donor­(pyridine) → p­(Sn) interaction in Sn­(^*t*−Bu^pdp^*t*–Bu^) **1**. The LUMO character of the stannylene changes from purely pyridine to (largely) p­(Sn) and the LUMO energy is significantly lowered (LUMO inversion). Consequently, the nature of lowest energy emissive triplet state changes from ^3^ILCT (pyrrolide → pyridine) to ^3^LMCT (pyrrolide → p­(Sn)). This topologically enabled lower ^3^LMCT energy shifts the phosphorescence from the green emission color of Sn­(bpep) to the red emission color of Sn­(^*t*−Bu^pdp^*t*–Bu^) **1**.

Apart from the in-plane pyridine-tin­(II) π-interaction in **1**, the accessible p­(Sn) orbital and the planarity of **1** enable coordination of donor solvents such as THF in solution and the possible formation of H aggregates in the solid state. The van-der-Waals dimer **[1···1]**, present in the solid state, leads to excitonic coupling with a brighter higher-energy and an almost dark lower-energy split level leading to a nonmonotonic emission intensity dependence on temperature.

This study provides a concept to rational ligand design for photoluminescent tin­(II) complexes by topological control of the frontier orbital energies and state characters. Future design concepts for phosphorescent stannylenes should additionally include steric shielding and rigidification to prevent σ-donor coordination to the Sn­(p) orbital, to avoid excitonic coupling invoked by aggregation via van-der-Waals forces and to mitigate (dissociative) large-amplitude molecular motions in the excited states reducing nonradiative decay.

## Experimental Section

### General Procedures

All reactions and measurements were performed under argon atmosphere unless otherwise noted. Gloveboxes (UniLab/MBraun – Ar 4.8, O_2_ < 0.1 ppm, H_2_O < 0.1 ppm) were used to store and weigh sensitive compounds for synthesis as well as to prepare samples that require absence of oxygen and water. The reagents were purchased from commercial suppliers (ABCR, Acros Organics, Alfa Aesar, Fischer Scientific and Sigma-Aldrich). Acetonitrile was dried and distilled from CaH_2_, diethyl ether and *n*-pentane from sodium and THF from potassium. Deuterated solvents were purchased from euriso-top and Deutero GmbH and were dried by the same procedures as above and stored over molecular sieve (3 Å). The compounds H_2_^*t*–Bu^pdp^*t*–Bu^ ^59^ and Sn­[N­(SiMe_3_)_2_] ^55,56,74^ were prepared according to literature procedures. Solution NMR spectra were recorded on a *Bruker* Avance DRX 400 spectrometer at 400.42 MHz (^1^H), 100.70 MHz (^13^C­{^1^H}) and 149.23 MHz (^119^Sn). The hydrodynamic radii *r*_H_ from the DOSY-derived diffusion coefficients *D* were determined with the Stokes–Einstein equation $r_{H}=\frac{k_{B}\cdot T}{6\pi \cdot \eta \cdot D}$ with the viscosity of benzene given as η = 0.6498 mPa s at 293 K.^75,76^ The ^119^Sn cross-polarization (CP) solid state (ss) NMR experiment was conducted on a Bruker DSX 400 MHz spectrometer at ^1^H frequency of 399.87 MHz and ^119^Sn frequency of 149.1 MHz using a 2.5 mm Bruker probe head at 20 kHz magic angle spinning (MAS). The initial 90° pulse was with a 4.0 μs length and a 4 s recycle delay was used. A ramped CP pulse (64–100%) with duration of 2 ms was used for recording the spectrum averaging 20 k transients. Two pulse phase modulation (TPPM) ^1^H decoupling scheme was used while acquiring the ^119^Sn NMR signal. The spectra were baseline-corrected, and a broadening of 200 Hz was applied. The ^119^Sn NMR shift was referenced to external tetramethyltin (SnMe_4_, δ = 0 ppm). All data were evaluated with the software MestReNova 12.0.4-22,023. All resonances are reported in ppm versus the solvent signal as internal standard (^1^H); (^13^C) NMR: THF-*d*_8_: δ = 1.72, 3.58; (25.31, 67.21) ppm or external standard for ^119^Sn NMR (SnMe_4_, δ = 0 ppm). (s) = singlet, (d) = doublet, (t) = triplet. Atmospheric pressure chemical ionization, APCI^+^ mass spectra were recorded on an Agilent 6545 QTOF-MS spectrometer by the central analytical facility of the Department of Chemistry of the Johannes Gutenberg University Mainz, Germany. The elemental analysis was performed by the Mikroanalytisches Labor Kolbe, c/o Fraunhofer Institut UMSICHT, Oberhausen, Germany. UV/vis absorption spectra were recorded on *a* Jasco V-770 spectrometer or a Cary*-5000* spectrometer from Agilent using *d* = 1.00 cm quartz cuvettes with a Schott valve. Steady-state emission spectra and photoluminescent decay curves were measured with a FLS1000 spectrometer from Edinburgh Instruments equipped with a cooled photomultiplier detector PMT-980 and a cooled NIR-sensitive photomultiplier detector N-G09 PMT-1700. A xenon arc lamp Xe2 (450 W) was used for excitation in steady-state measurements. Time-resolved luminescence experiments were recorded in the multichannel scaling mode employing a pulsed diode laser VPL-450 (λ_exc_ = 451.3 nm) as excitation source. Single and biexponential lifetime decays were evaluated with the software Fluoracle provided by Edinburgh Instruments. Measurements at low temperature were carried out using a liquid nitrogen-cooled cryostat Optistat *DN* from Oxford Instruments. Absolute luminescence quantum yields Φ were determined using the MicrostatN from Oxford Instruments combined with the Cryosphere from Edinburgh Instruments. Relative uncertainty of Φ is estimated to be ±20%. Density functional theory calculations were performed with the Orca 5.0.4. Program package.^77,78^ Geometry optimizations were performed using (un)­restricted Kohn–Sham orbitals DFT (RKS or UKS) and the B3LYP functional^79–81^ in combination with the ZORA-def2-TZVPP basis as recontracted Ahlrich’s basis set def2-TZVPP^82^ or the SARC-ZORA-TZVPP basis^83–87^ with the auxiliary basis SARC/J^83–86^ as decontracted def2/J^88^ basis up to Kr to use the zeroth order regular approximation to describe relativistic effects in all calculations (keyword *ZORA*).^89,90^ Tight convergence criteria were chosen for (TD)­DFT calculations (keywords *tightscf* and *tightopt*). The geometry optimization of the T_2_ state was performed by TDDFT using the LibXC^91^ library for the B3LYP functional^92^ (keyword *LibXC­(B3LYP)*) with the *followiroot true* keyword. The validity of the B3LYP functional in SOC-TDDFT calculations of **[1···1]** ^**H**^ was tested in comparative calculations with the CAM-B3LYP,^93^ M06-2X,^94,95^ and TPPSh^96–98^ functionals. All DFT calculations make use of the resolution of identity (Split-RI-J) approach for the Coulomb term in combination with the chain-of-spheres approximation for the exchange term (keyword *RIJCOSX*).^99–102^ To account for solvent effects, a conductor-like screening model (keyword *CPCM*(hexane)) modeling hexane was used in all calculations.^103,104^ Atom-pairwise dispersion correction was performed with the Becke-Johnson damping scheme (keyword *D3BJ*).^105,106^ A numerical frequency calculation confirmed that the optimized geometry corresponds to a minimum structure. Explicit solvent molecules were not taken into account. The charge transfer number analyses of the TDDFT calculated transitions were done using TheoDORE.^107,108^ Twenty spin-allowed and spin-forbidden and 50 spin-allowed transitions were calculated by TDDFT with and without the keyword *triplets true*. SOC-TDDFT calculations were performed using the additional keywords *DoSOC true*, *TDA false* and *RI-SOMF­(1X)*.^109^ The QTAIM analysis was performed with *Avogadro 2*.0.0 ^110^ (visualization) and Multiwfn 3.8 (quantitative values).^111,112^ All calculations were computed on the supercomputer Elwetritsch with advisory services offered by the RPTU Kaiserslautern-Landau (https://hpc.rz.rptu.de), which is a member of the AHRP. All coordinates of the optimized structures are provided in an additional xyz file. The crystals for single crystal structure determinations were collected in a glovebox and covered with paraffine oil for IR spectroscopy. The crystals under oil were selected with a microscope at ambient conditions and mounted with a nylon loop on the goniometer head. Intensity data were collected with a STOE IPDS-2T or a STOE STADIVARI diffractometer from STOE & CIE GmbH with an Oxford cooling device using Mo–Kα radiation (λ = 0.71073 Å). The diffraction frames were integrated using the STOE X-Area^113^ software package and were corrected for absorption with MULABS^114^ of the PLATON^115^ software package or with STOE X-Red^116^ or STOE LANA^122,117^ of the STOE X-Area^113^ software package. The structures were solved with SHELXT^118^ and refined by the full-matrix method based on *F* ^2^ using SHELXL^119^ of the SHELX^120^ software package and the ShelXle^121^ graphical interface. All non-hydrogen atoms were refined anisotropically while the positions of all hydrogen atoms were generated with appropriate geometric constraints and allowed to ride on their respective parent atoms with fixed isotropic thermal parameters. Crystallographic data for the structures reported in this paper has been deposited with the Cambridge Crystallographic Data Centre as supplementary publication no. CCDC-2531627 (**1**@120 K), CCDC-2531628 (**1**@100 K), CCDC-2531629 (**1**@150 K), CCDC-2531630 (**1**@175 K), CCDC-2531631 (**1**@225 K), CCDC-2531632 (**2**). Crystallographic data is listed in the Supporting Information.

### Synthesis of Sn­(^*t*−Bu^pdp^*t*–Bu^) **1**

Sn­[(N­(SiMe_3_)_2_]_2_ (419 mg, 0.95 mmol, 1.0 equiv) in diethyl ether (4 mL) was added to a stirred solution of H_2_^*t*–Bu^pdp^*t*–Bu^ (413 mg, 0.95 mmol, 1.00 equiv) in acetonitrile (12 mL). After stirring for 15 h at 293 K, the formed precipitate was separated from the solution with a centrifuge (3500 rounds min^–1^, 15 min), washed with acetonitrile (3 × 3 mL) and dried under reduced pressure. **1** (235 mg, 44%) was obtained as a red powder. Crystals suitable for XRD analysis were obtained by slow evaporation of a concentrated acetonitrile solution of **1**. ^**1**^ **H NMR** (THF-*d*_8_): δ = 7.51 (t, ^3^ *J*_HH_ = 8.0, 1H, H^1^), 7.29 (d, ^3^ *J*_HH_ = 8.0, 2H, H^2^), 6.03 (s, 2H, H^10^), 1.44 (s, 18H, H^13,14^), 1.43 (s, 18H, H^7–9^) ppm. ^**1**^ **H NMR** (C_6_D_6_): δ = 7.25 (d, ^3^ *J*_HH_ = 8.0, 2H, H^2^), 6.95 (t, ^3^ *J*_HH_ = 8.0, 1H, H^1^), 6.48 (s, 2H, H^10^), 1.61 (s, 18H, H^13,14^), 1.46 (s, 18H, H^7–9^) ppm. ^**13**^ **C­{** ^**1**^ **H} NMR** (THF-*d*_8_): δ = 153.3 (s, C^9^), 152.7 (s, C^11^), 137.8 (s, C^1^), 137.0 (s, C^5^), 130.5 (s, C^4^), 114.8 (s, C^2^), 109.3 (s, C^10^), 34.0 (s, C^12^), 33.2 (s, C^13–15^), 32.4 (s, C^7–9^), 32.2 (s, C^6^) ppm. ^**119**^ **Sn NMR** (THF-*d*_8_): δ = −466.5 ppm. ^**119**^ **Sn NMR** (solid): δ = −101.6 ppm. **MS** (APCI^+^; CH_3_CN): *m*/*z* (%) = 433.35 (100) [H_2_^*t*–Bu^pdp^*t*–Bu^ + H]^+^, 552.24 (11) [**1** + H] ^+^, 1119.48 (20) [2 **1** + H_2_O + H] ^+^. Elemental analysis calcd for C_29_H_41_N_3_Sn: C, 63.29; H, 7.51; N, 7.63. Found: C, 63.04; H, 7.46; N, 7.58.

## Supplementary Material





## Data Availability

All the raw data from this manuscript have been uploaded to Zenodo and are freely available via the DOI 10.5281/zenodo.20089734.

## Notes

Cartesian coordinates of optimized geometries from quantum-chemical calculations (XYZ)
Analytical and spectroscopic data, results of quantum chemical calculations (PDF)
