# Supplementary material for: Ligand Topology Tunes Energy and Character of Emissive Triplet States in Stannylenes
Source: Inorg Chem. 2026 May 28;65(22):12409–21. doi: 10.1021/acs.inorgchem.6c01229 (PMC13250984; doi:10.1021/acs.inorgchem.6c01229)
Supplement: Supplementary file 2 [file ic6c01229_si_002.pdf]

Supporting Information File
Ligand Topology Tunes Energy and Character of Emissive Triplet States in Stannylenes

Philipp Sikora, Robert Naumann, Katja Heinze* and Christoph Förster*

Department of Chemistry, Johannes Gutenberg University Mainz, Duesbergweg 10–14, 55128 Mainz (Germany)

E-Mails: katja.heinze@uni-mainz.de, christoph.foerster@uni-mainz.de

Crystallographic Data of Sn($^{t\text{-Bu}}$pdp$^{t\text{-Bu}}$) **1**

$C_{29}H_{41}N_3Sn$ (550.34); triclinic; $P\bar{1}$; *a* = 10.347(2) Å, *b* = 10.991(2) Å, *c* = 13.383(3) Å; α = 71.82(3)°; β = 88.06(3)°; γ = 67.08(3)°; *V* = 1325.2(6) Å^3; *Z* = 2; density, calcd. = 1.379 g cm^{-3}, *T* = 120(2) K, μ = 0.985 mm^{-1}; *F*(000) = 572; crystal size 0.640 × 0.550 × 0.430 mm^3; θ = 2.811 to 27.863 deg.; −13≤h≤13, −14≤k≤14, −16≤l≤17; rfln collected = 11914; rfln unique = 6284 [*R*(int) = 0.0200]; completeness to θ = 25.242 deg.: 99.6%; absorption correction: Semi-empirical from equivalents; max. and min. transmission 0.4841 and 0.3993; data 6284; restraints 0, parameters 310; goodness-of-fit on F^2 = 1.127; final indices [$I > 2\sigma(I)$] R_1 = 0.0231, wR_2 = 0.0564; *R* indices (all data) R_1 = 0.0254, wR_2 = 0.0575; largest diff. peak and hole 0.519 and −0.296 e Å^{-3}.

Crystallographic Data of Sn($^{t\text{-Bu}}$pdp$^{t\text{-Bu}}$) **1** from variable-temperature measurements

100 K: $C_{29}H_{41}N_3Sn$ (550.34); triclinic; $P\bar{1}$; *a* = 10.3515(6) Å, *b* = 10.9903(6) Å, *c* = 13.3717(7) Å; α = 71.796(4)°; β = 88.045(4)°; γ = 67.127(4)°; *V* = 1324.84(13) Å^3; *Z* = 2; density, calcd. = 1.380 g cm^{-3}, *T* = 100(2) K, μ = 0.985 mm^{-1}; *F*(000) = 572; crystal size 0.390 × 0.310 × 0.210 mm^3; θ = 2.127 to 32.940 deg.; −15≤h≤15, −15≤k≤16, −18≤l≤20; rfln collected = 22540; rfln unique = 8386 [*R*(int) = 0.0289]; completeness to θ = 25.242 deg.: 99.0%; absorption correction: integration; max. and min. transmission 0.9054 and 0.6744; data 8386; restraints 0, parameters 310; goodness-of-fit on F^2 = 1.034; final indices [$I > 2\sigma(I)$] R_1 = 0.0315, wR_2 = 0.0746; *R* indices (all data) R_1 = 0.0368, wR_2 = 0.0753; largest diff. peak and hole 1.308 and −1.497 e Å^{-3}.

150 K: $C_{29}H_{41}N_3Sn$ (550.34); triclinic; $P\bar{1}$; *a* = 10.3590(7) Å, *b* = 11.0232(7) Å, *c* = 13.4186(8) Å; α = 71.581(5)°; β = 87.946(5)°; γ = 67.106(5)°; *V* = 1332.58(16) Å^3; *Z* = 2; density, calcd. = 1.372 g cm^{-3}, *T* = 150(2) K, μ = 0.980 mm^{-1}; *F*(000) = 572; crystal size 0.390 × 0.310 × 0.210 mm^3; θ = 2.123 to 32.954 deg.; −15≤h≤15, −15≤k≤16, −19≤l≤20; rfln collected = 22791; rfln unique = 8434 [*R*(int) = 0.0304]; completeness to θ = 25.242 deg.: 99.2%; absorption correction: integration; max. and min. transmission 0.9062 and 0.6797; data 8434; restraints 0, parameters 310; goodness-of-fit on F^2 = 1.027; final indices [$I > 2\sigma(I)$] R_1 = 0.0319, wR_2 = 0.0737; *R* indices (all data) R_1 = 0.0379, wR_2 = 0.0744; largest diff. peak and hole 0.981 and −1.382 e Å^{-3}.

175 K: $C_{29}H_{41}N_3Sn$ (550.34); triclinic; $P\bar{1}$; *a* = 10.3695(6) Å, *b* = 11.0407(6) Å, *c* = 13.4415(8) Å; α = 71.443(4)°; β = 87.935(5)°; γ = 67.100(4)°; *V* = 1337.07(14) Å^3; *Z* = 2; density, calcd. = 1.367 g cm^{-3}, *T* = 175(2) K, μ = 0.976 mm^{-1}; *F*(000) = 572; crystal size 0.390 × 0.310 × 0.210 mm^3; θ = 2.122 to 32.916 deg.; −15≤h≤15, −15≤k≤16, −19≤l≤20; rfln collected = 22992; rfln unique = 8458 [*R*(int) = 0.0296]; completeness to θ = 25.242 deg.: 99.0%; absorption correction: integration; max. and min. transmission 0.9061 and 0.6789; data 8458; restraints 0, parameters 310; goodness-of-fit on F^2 = 1.017; final indices [$I > 2\sigma(I)$] R_1 = 0.0311, wR_2 = 0.0708; *R* indices (all data) R_1 = 0.0381, wR_2 = 0.0715; largest diff. peak and hole 0.998 and −1.238 e Å^{-3}.

225 K: $C_{29}H_{41}N_3Sn$ (550.34); triclinic; $P\bar{1}$; *a* = 10.3748(3) Å, *b* = 11.0785(3) Å, *c* = 13.4980(4) Å; α = 71.213(2)°; β = 88.013(2)°; γ = 67.048(2)°; *V* = 1345.11(7) Å^3; *Z* = 2; density, calcd. = 1.359 g cm^{-3}, *T* = 225(2) K, μ = 0.971 mm^{-1}; *F*(000) = 572; crystal size 0.390 × 0.310 × 0.210 mm^3; θ = 2.119 to 33.843 deg.; −16≤h≤16, −16≤k≤16, −19≤l≤21; rfln collected = 28066; rfln unique = 9321 [*R*(int) = 0.0287]; completeness to θ = 25.242 deg.: 99.8%; absorption correction: integration; max. and min. transmission 0.9073 and 0.6798; data 9321; restraints 0, parameters 310; goodness-of-fit on F^2 = 1.013; final indices [$I > 2\sigma(I)$] R_1 = 0.0313, wR_2 = 0.0767; *R* indices (all data) R_1 = 0.0372, wR_2 = 0.0783; largest diff. peak and hole 1.076 and −1.157 e Å^{-3}.

Crystallographic Data of $Sn_2(^{t\text{-Bu}}pdp^{t\text{-Bu}})_2$ **2**

Crystals for XRD analysis were obtained from a solution of a mixture of **1** and **2** in diethyl ether after storing at –30 °C for six weeks.

$C_{62}H_{92}N_6OSn_2$ (1174.79); triclinic; $P\bar{1}$; *a* = 14.254(3) Å, *b* = 14.370(3) Å, *c* = 15.023(3) Å; α = 82.07(3)°; β = 87.03(3)°; γ = 83.87(3)°; *V* = 3028.0(11) Å^3; *Z* = 2; density, calcd. = 1.288 g cm^{-3}, *T* = 120(2) K, μ = 0.868 mm^{-1}; *F*(000) = 1228; crystal size 0.170 × 0.083 × 0.020 mm^3; θ = 2.504 to 27.966 deg.; −18≤h≤17, −18≤k≤18, −19≤l≤19; rfln collected = 52878; rfln unique = 14423 [*R*(int) = 0.1600]; completeness to θ = 25.242 deg.: 99.9%; absorption correction: semi-empirical from equivalents; max. and min. transmission 1.19353 and 0.82302; data 14423; restraints 0, parameters 666; goodness-of-fit on F^2 = 1.089; final indices [$I > 2\sigma(I)$] R_1 = 0.0627, wR_2 = 0.1055; *R* indices (all data) R_1 = 0.1182, wR_2 = 0.1263; largest diff. peak and hole 0.795 and −1.068 e.Å^{-3}.

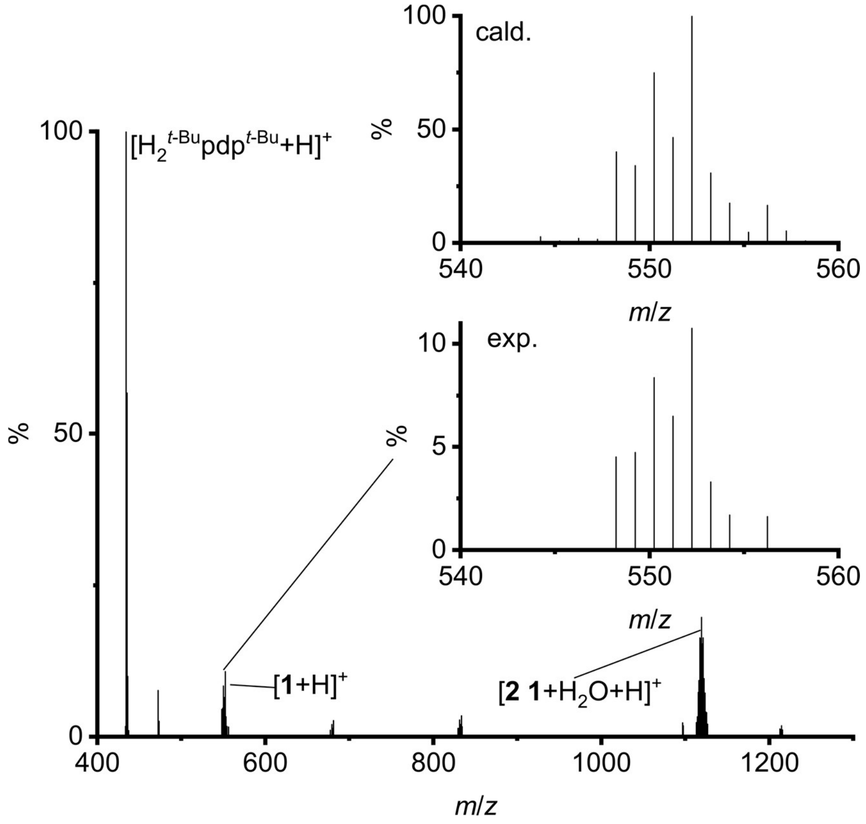

Figure S1. APCI$^+$ mass spectrum of **1** in acetonitrile with insets of the experimentally found and calculated isotope pattern for **[1+H]$^+$**.

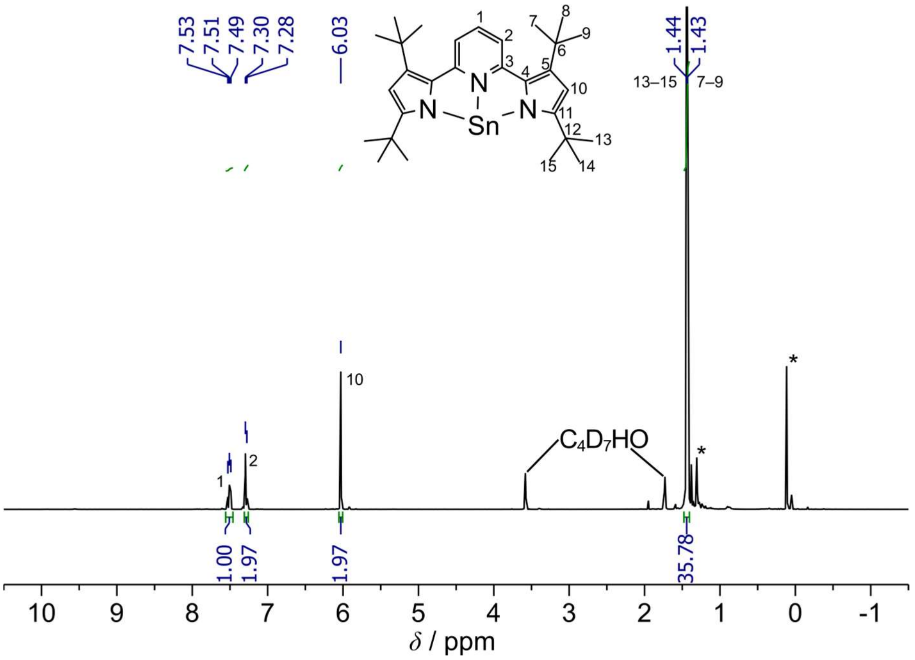

Figure S2. ^{1}H NMR spectrum of **1(thf-*d*8)2** in tetrahydrofuran-*d*8 with atom numbering for the assignment. The asterisks denote resonances of silicon grease and small amounts of hydrolysis products.

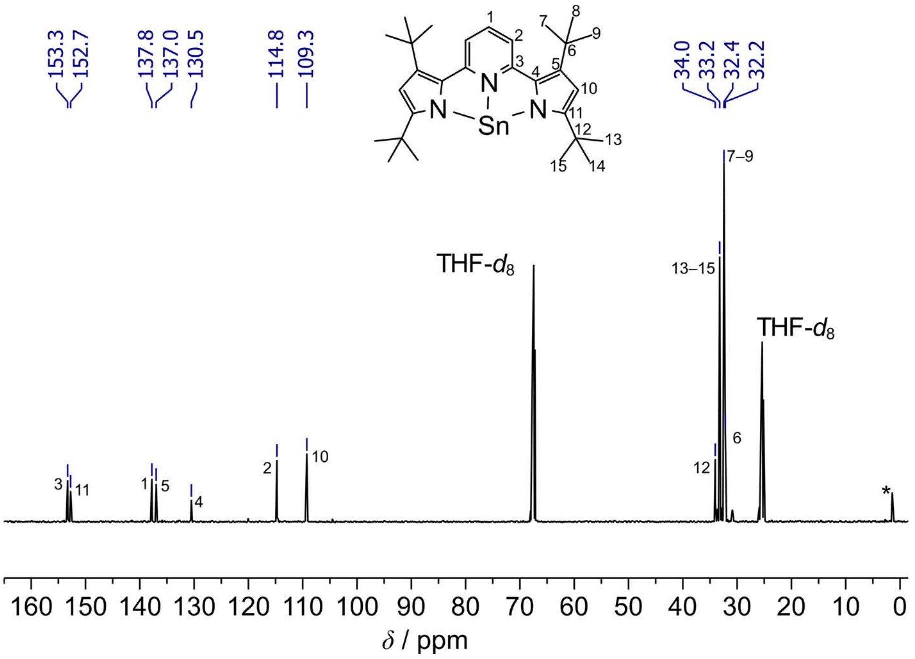

Figure S3. $^{13}C\{^{1}H\}$ NMR spectrum of **1(thf-*d*8)2** in tetrahydrofuran-*d*8 with atom numbering for the assignment. The asterisk denotes resonance of silicon grease.

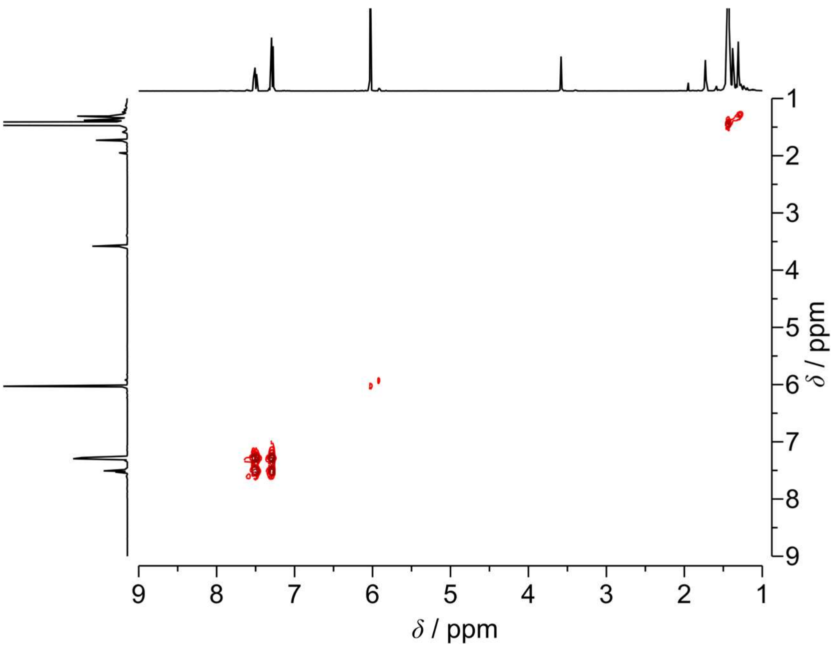

Figure S4. $^{1}H,^{1}H$ COSY NMR spectrum of **1(thf-*d*$_8$)$_2$** in tetrahydrofuran-*d*$_8$.

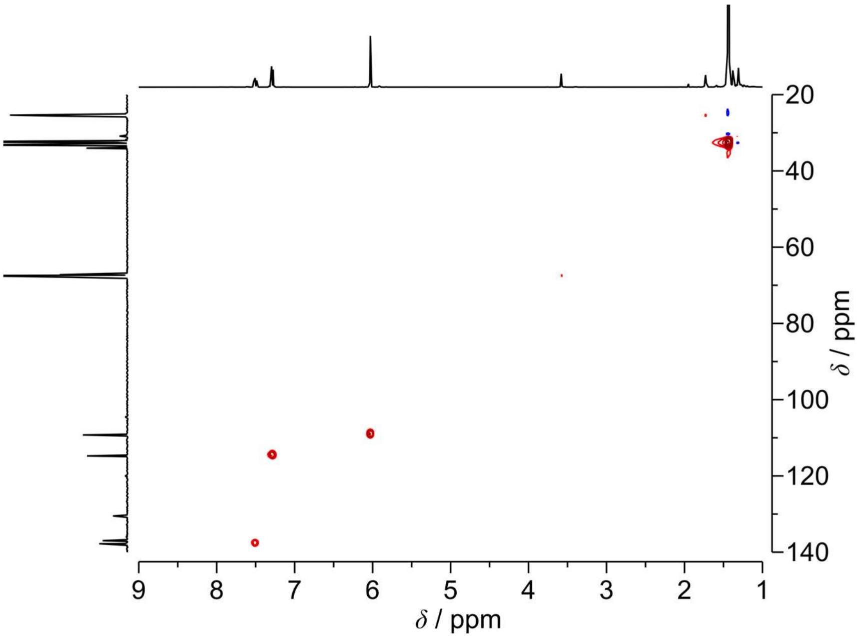

Figure S5. $^{13}C,^{1}H$ HSQC NMR spectrum of **1(thf-*d*$_8$)$_2$** in tetrahydrofuran-*d*$_8$.

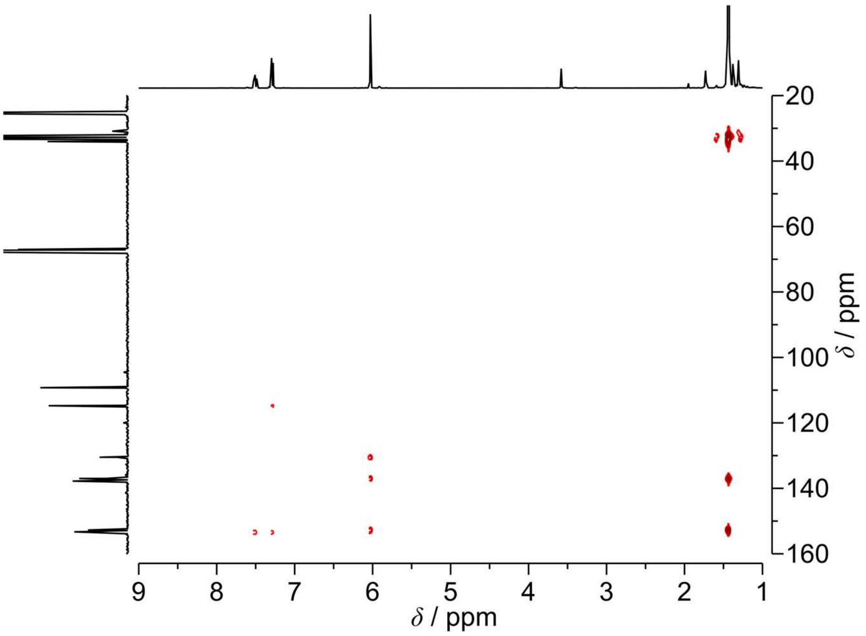

Figure S6. $^{13}C,^{1}H$ HMBC NMR spectrum of **1(thf-*d*$_8$)$_2$** in tetrahydrofuran-*d*$_8$.

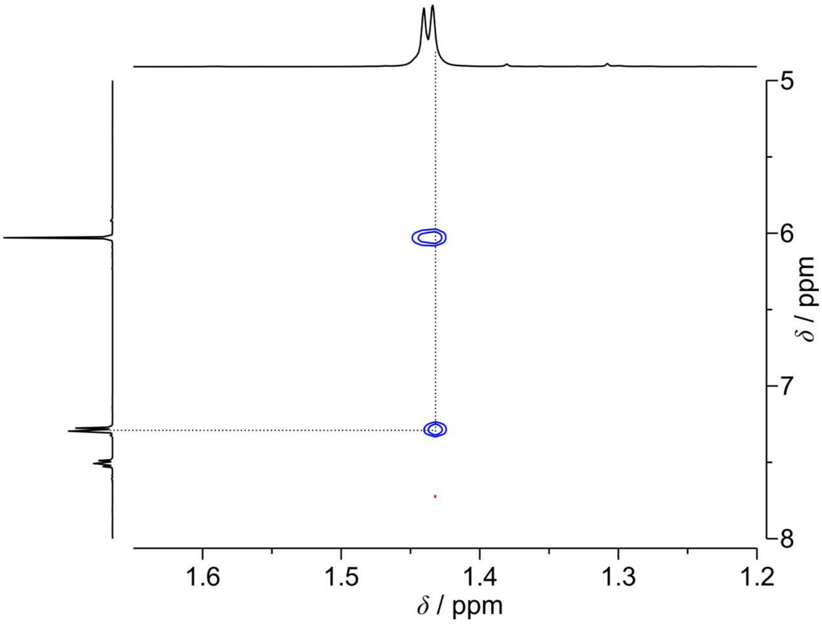

Figure S7. Part of the $^{1}H,^{1}H$ NOESY NMR spectrum of **1(thf-*d*$_8$)$_2$** in tetrahydrofuran-*d*$_8$ for assignment of the *tert*-butyl resonances

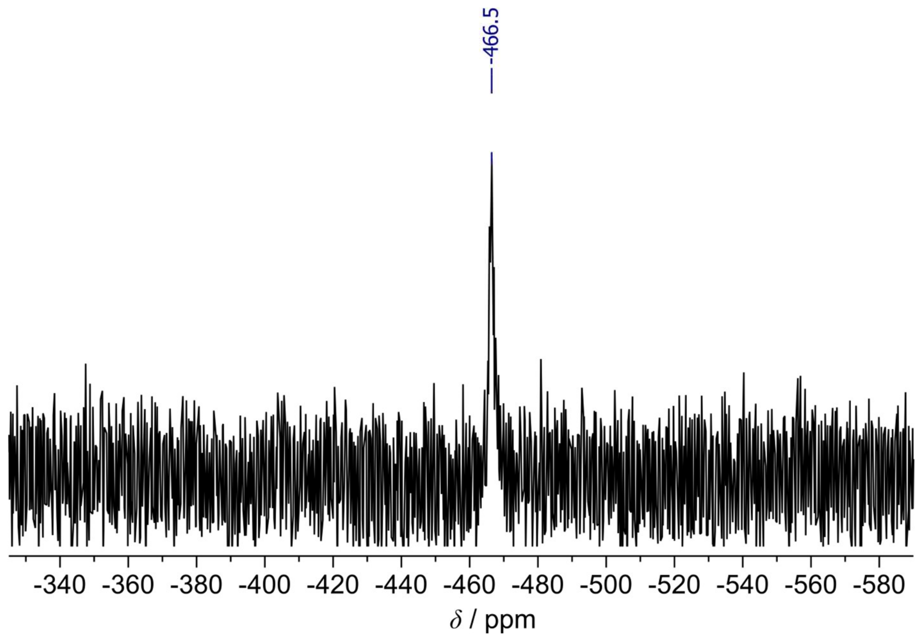

Figure S8. ^{119}Sn NMR spectrum of **1(thf-*d*$_8$)$_2$** in tetrahydrofuran-*d*$_8$.

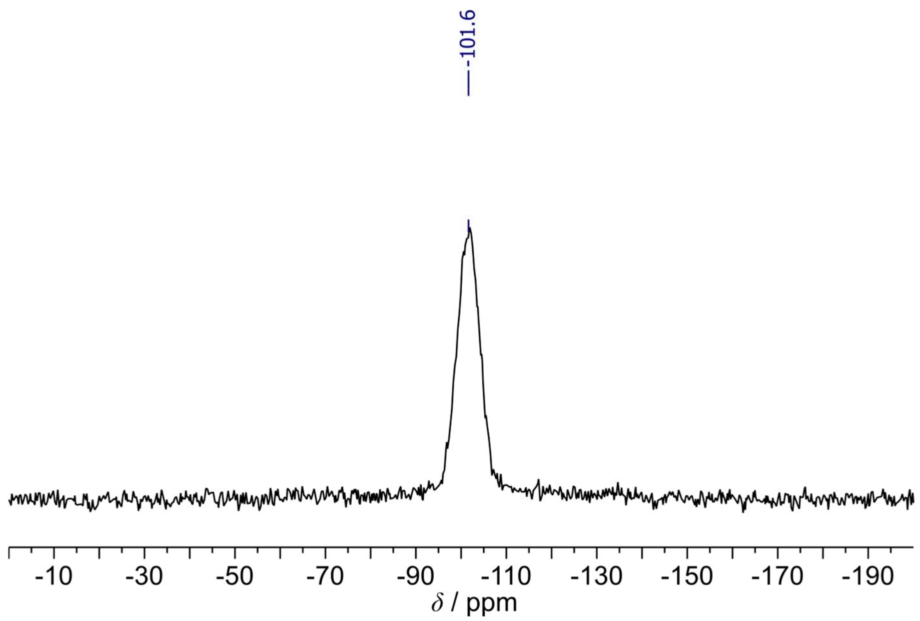

Figure S9. ^{119}Sn CP-MAS NMR spectrum of **1** in the solid state.

Table S1. Selected structural parameters of **1** in Å and degrees [°] of the molecular structure of **1** from SC-XRD of two crystals at 120 K and in the series 100–225 K and DFT calculated geometry optimized structures of **1** in the S_0, T_1 andT_2 states.

	**1** (XRD 120 K)	S_0 (DFT)	T_1 (DFT)	T_2 (DFT)
Sn−N^{py}	2.1680(16)	2.197	2.247	2.266
Sn−N^{pyr1}	2.2197(18)	2.247	2.382	2.339
Sn–N^{pyr2}	2.1967(15)	2.238	2.381	2.334
N^{pyr1}−Sn−N^{pyr2}	117.07(6)	117.04	141.06	136.08
N^{py}−Sn−N^{pyr1}	72.73(6)	71.87	70.49	70.07
N^{py}−Sn−N^{pyr2}	72.36(6)	71.82	70.57	70.39
angle sum	262.16	260.73	282.12	276.54

	**1** (XRD 100K)	**1** (XRD 150K)	**1** (XRD 175K)	**1** (XRD 225K)
Sn−N^{py}	2.1697(16)	2.1679(16)	2.1676(15)	2.1679(14)
Sn−N^{pyr1}	2.2231(15)	2.2237(15)	2.2228(14)	2.2232(13)
Sn–N^{pyr2}	2.1922(15)	2.1935(15)	2.1920(15)	2.1972(13)
N^{pyr1}−Sn−N^{pyr2}	117.04(6)	117.05(6)	117.09(5)	117.06(5)
N^{py}−Sn−N^{pyr1}	72.71(6)	72.58(6)	72.58(5)	72.59(5)
N^{py}−Sn−N^{pyr2}	72.44(6)	72.51(5)	72.56(5)	72.47(5)
angle sum	262.19	262.14	262.23	262.12

Table S2. Selected structural parameters in Å and degrees [°] of the molecular structure of **2** from SC-XRD at 120 K and from DFT geometry optimization.

	**1** (XRD 120 K)	S_0 (DFT)
Sn−N^{py}	2.224(5)	2.270
Sn−N^{pyr1}	2.200(4)	2.237
Sn–N^{pyr2}	2.165(4)	2.223
Sn'–$N^{py'}$	2.228(4)	2.270
Sn'–$N^{pyr1'}$	2.190(4)	2.237
Sn'–$N^{pyr2'}$	2.177(4)	2.224
N^{pyr1}−Sn−N^{pyr2}	99.75(16)	100.16
N^{py}−Sn−N^{pyr1}	73.76(16)	72.20
N^{py}−Sn−N^{pyr2}	97.82(17)	98.04
$N^{pyr1'}$−Sn'−$N^{pyr2'}$	99.18(16)	99.83
$N^{py'}$−Sn'−$N^{pyr1'}$	73.16(16)	72.20
$N^{py'}$−Sn'−$N^{pyr2'}$	98.82(16)	98.00

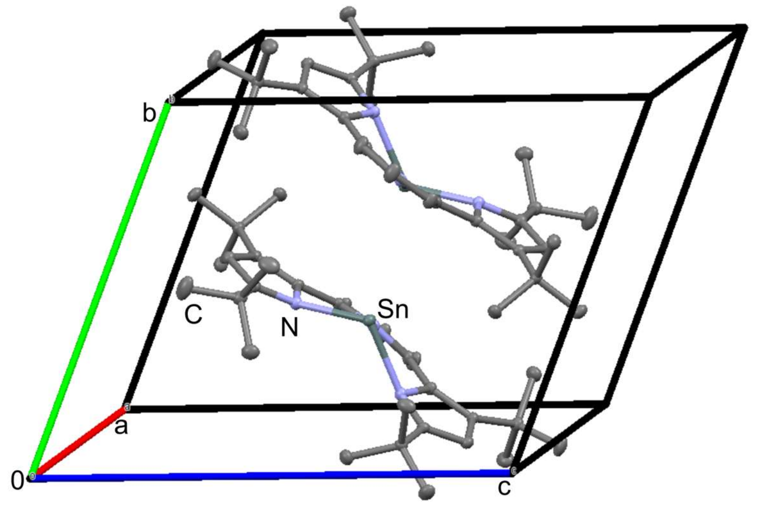

Figure S10. Unit cell of **1** in the solid state (triclinic space group $P\bar{1}$) showing the van-der-Waals pairs **[1···1]**. Hydrogen atoms omitted, thermal ellipsoids at 50% probability level.

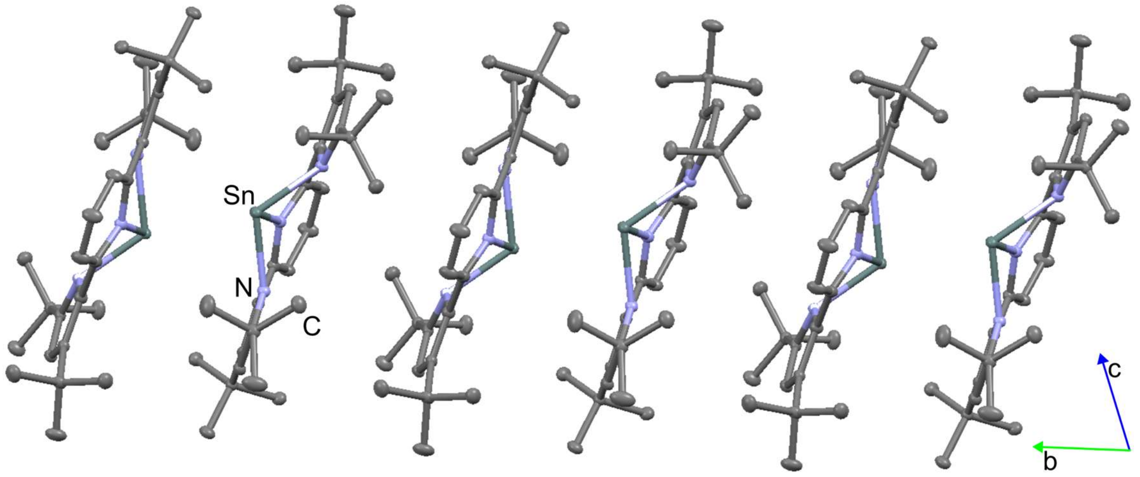

Figure S11. Selected stack of van-der-Waals pairs **[1···1]** along the *b* axis in the solid state. Hydrogen atoms omitted, thermal ellipsoids at 50% probability level.

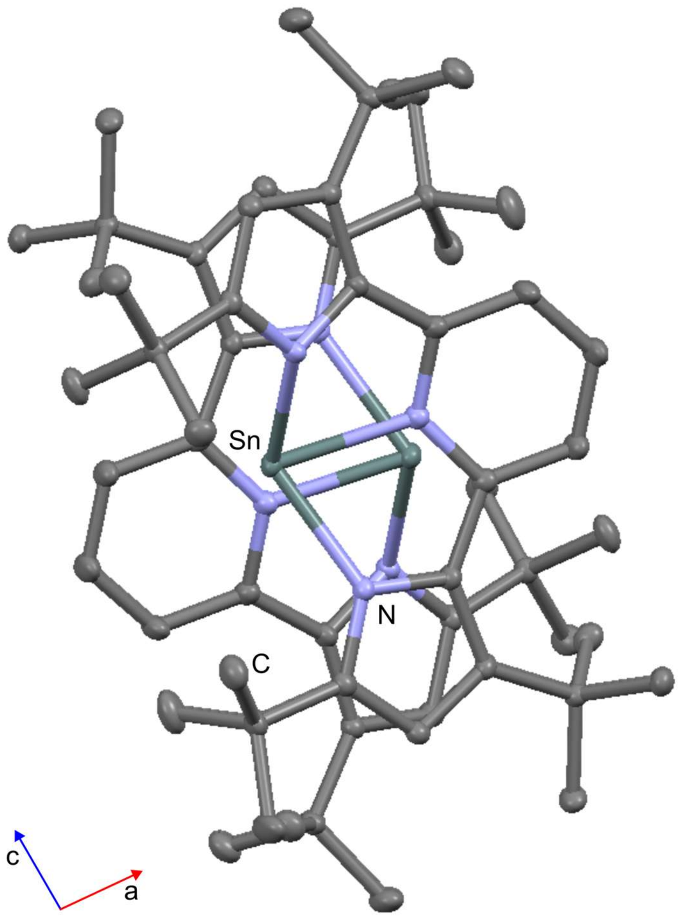

Figure S12. View of van-der-Waals pair **[1···1]** orthogonal along the *b* axis. Hydrogen atoms omitted, thermal ellipsoids at 50% probability level.

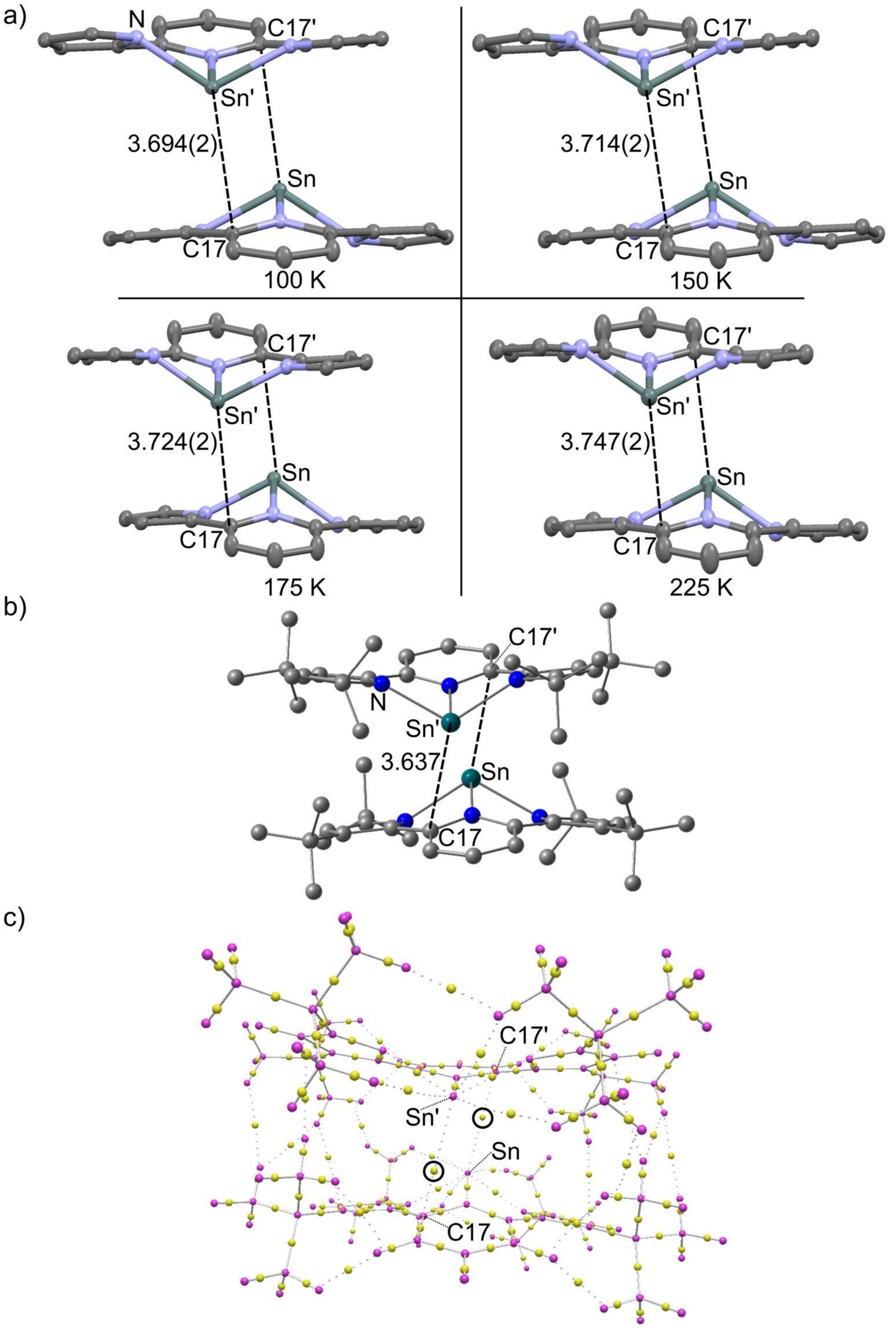

Figure S13. Van-der-Waals dimers **[1···1]** from variable-temperature SC-XRD analyses highlighting the Sn···C17' contacts with distances given in Å. Hydrogen atoms and *tert*-butyl substituents groups omitted, thermal ellipsoids at 50% probability level. b) Geometry optimized molecular structure of **[1···1]** from DFT calculations (CPCM(hexane)-RIJCOSX-B3LYP-D3BJ-ZORA-SARC/J-ZORA-def2-TZVPP/SARC-ZORA-TZVPP(Sn)). Hydrogen atoms omitted. c) QTAIM analysis with nuclear critical points (purple), bond critical points (yellow), electron density paths (dotted lines), and covalent interaction paths (solid lines) of **[1···1]**. Bond critical points between the Sn$^{(')}$···C17$^{(')}$ contacts highlighted with circles at (9.399, 6.757, 5.546) and (5.683, 7.709, 7.105) Å with electron density ρ = 0.00711 a.u., Laplacian of electron density $\nabla^2\rho$ = 0.0164 a.u., Lagrangian kinetic energy $G(\boldsymbol{r})$ = 0.00350 a.u., potential energy density $V(\boldsymbol{r})$ = –0.00290 a.u., and energy density $H(\boldsymbol{r})$ = 0.000600 a.u..

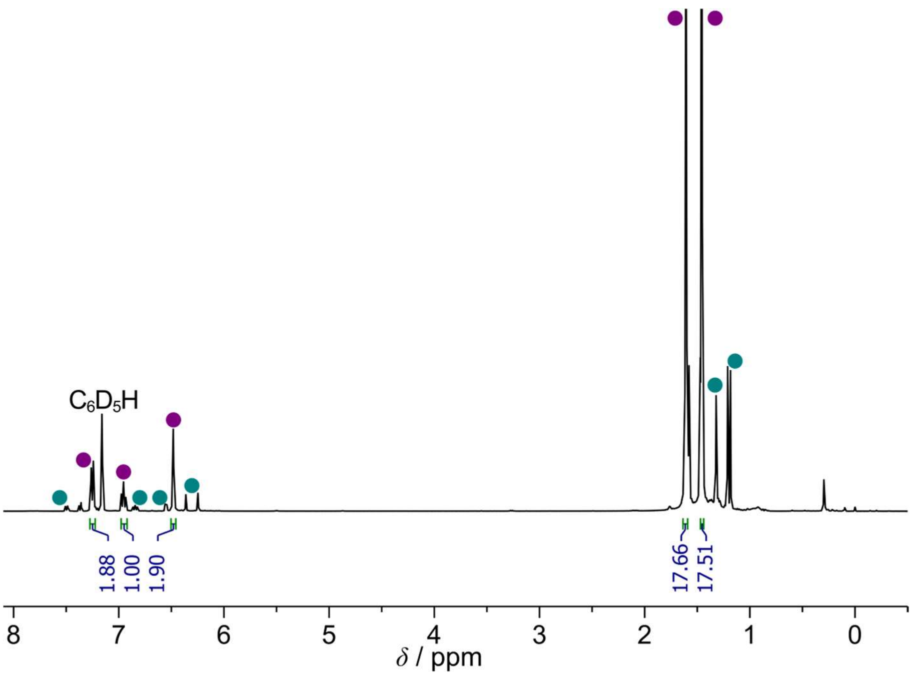

Figure S14. ^{1}H NMR spectrum of a mixture of **1** and **2** (1:0.125 ratio) in C_6D_6 after 40 minutes of sample preparation from solid **1** at 293 K (c = 48 mM). Resonances of **1** and **2** marked with purple and turquoise circles, respectively.

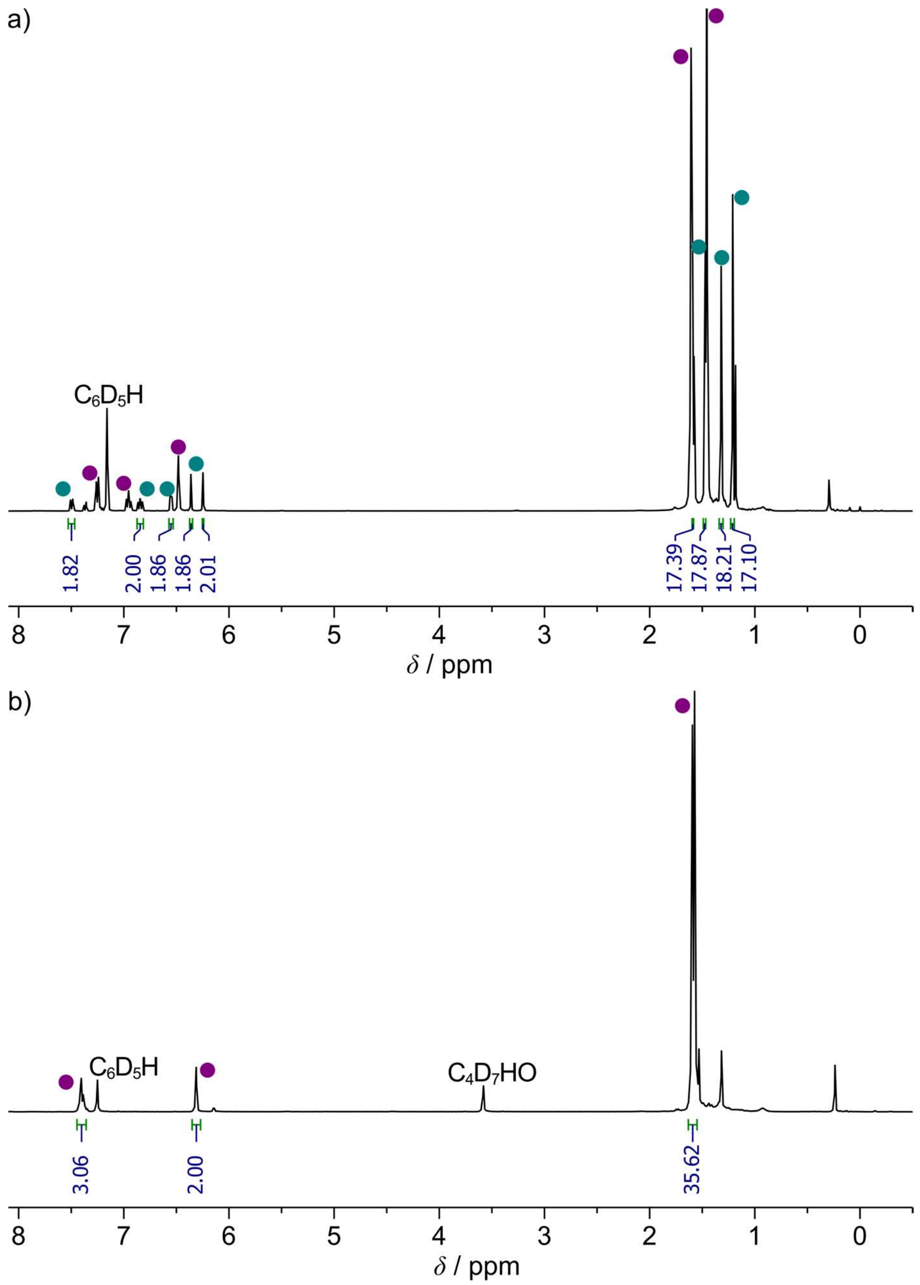

Figure S15. ^{1}H NMR spectrum of a) a mixture of **1** and **2** (1:0.42 ratio) in C_6D_6 after six hours of sample preparation from solid **1** at 293 K (c = 48 mM) and b) with addition of 0.25 mL THF-d_8 (in 0.5 mL C_6D_6) after three days of sample preparation from solid **1** at 293 K. Resonances of **1** and **2** marked with purple and turquoise circles, respectively.

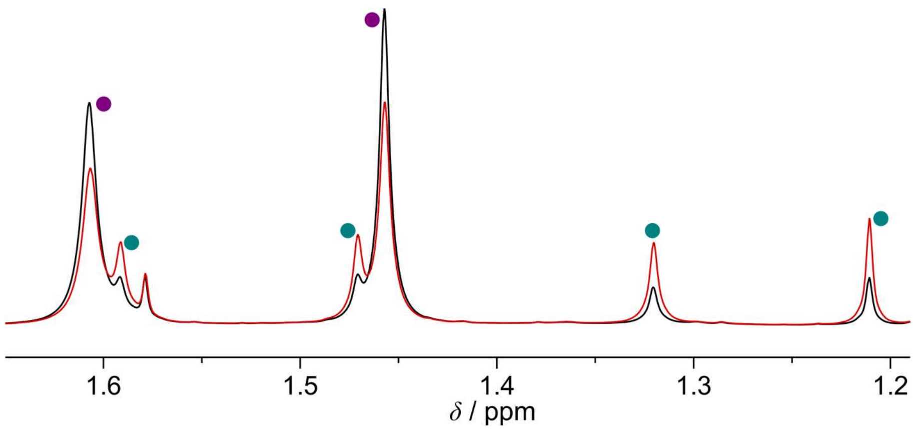

Figure S16. Comparison of the aliphatic region in the ^{1}H NMR spectra of a mixture of **1** and **2** in C_6D_6 after 40 minutes (black) and after six hours (red) of sample preparation from solid **1** at 293 K (c = 48 mM). Resonances of **1** and **2** marked with purple and turquoise circles, respectively

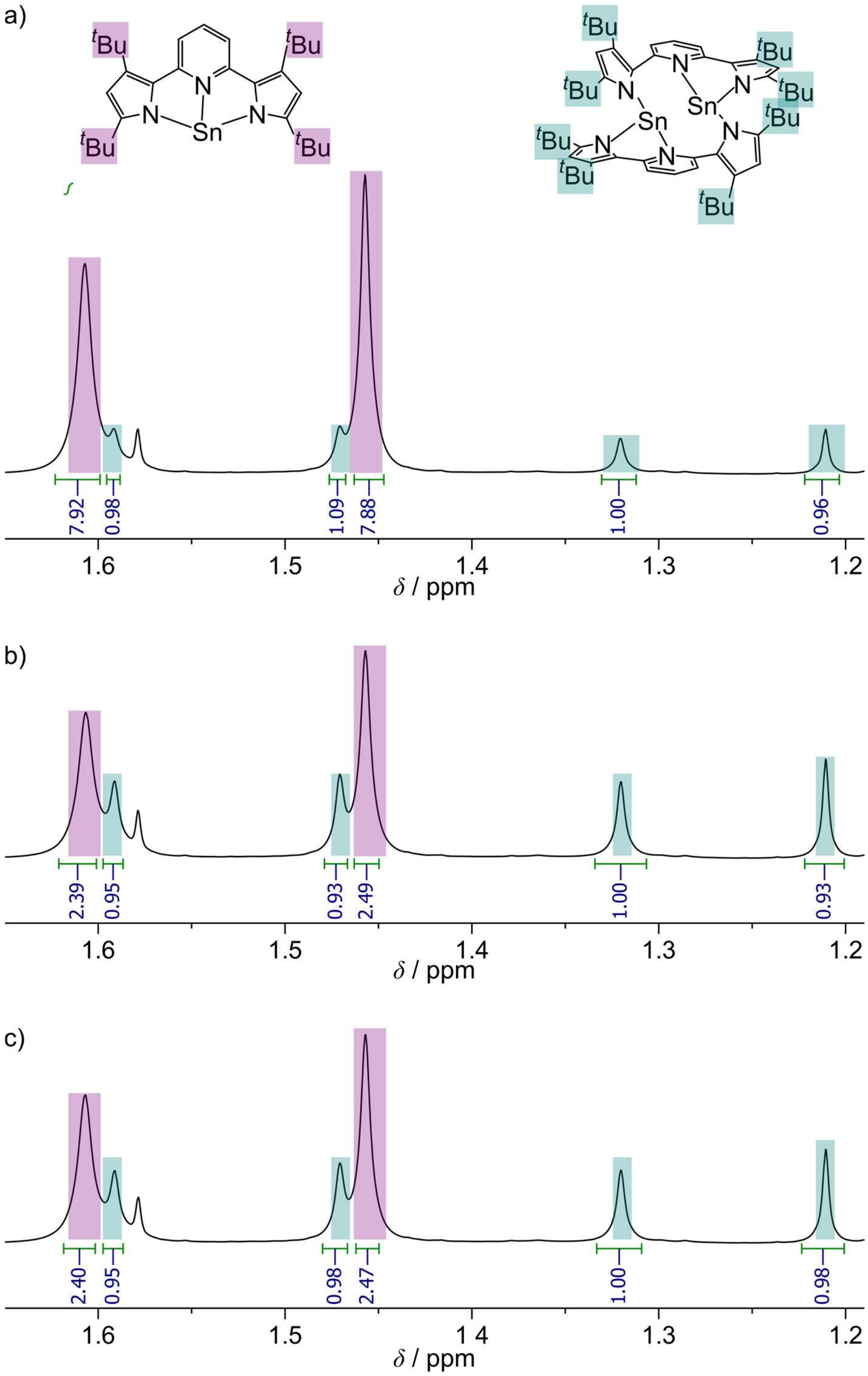

Figure S17. Integrated resonances in the aliphatic region in the ^{1}H NMR spectrum of a mixture of **1** and **2** after a) 40 min of sample preparation (1:0.125 ratio), b) after six hours of sample preparation (1:0.42 ratio) and c) after twelve hours of sample preparation (indicating no further changes and the established equilibrium between **1** and **2**) in C_6D_6 from solid **1** at 293K (c = 48 mM). Resonances of **1** and **2** marked with purple and turquoise bars, respectively.

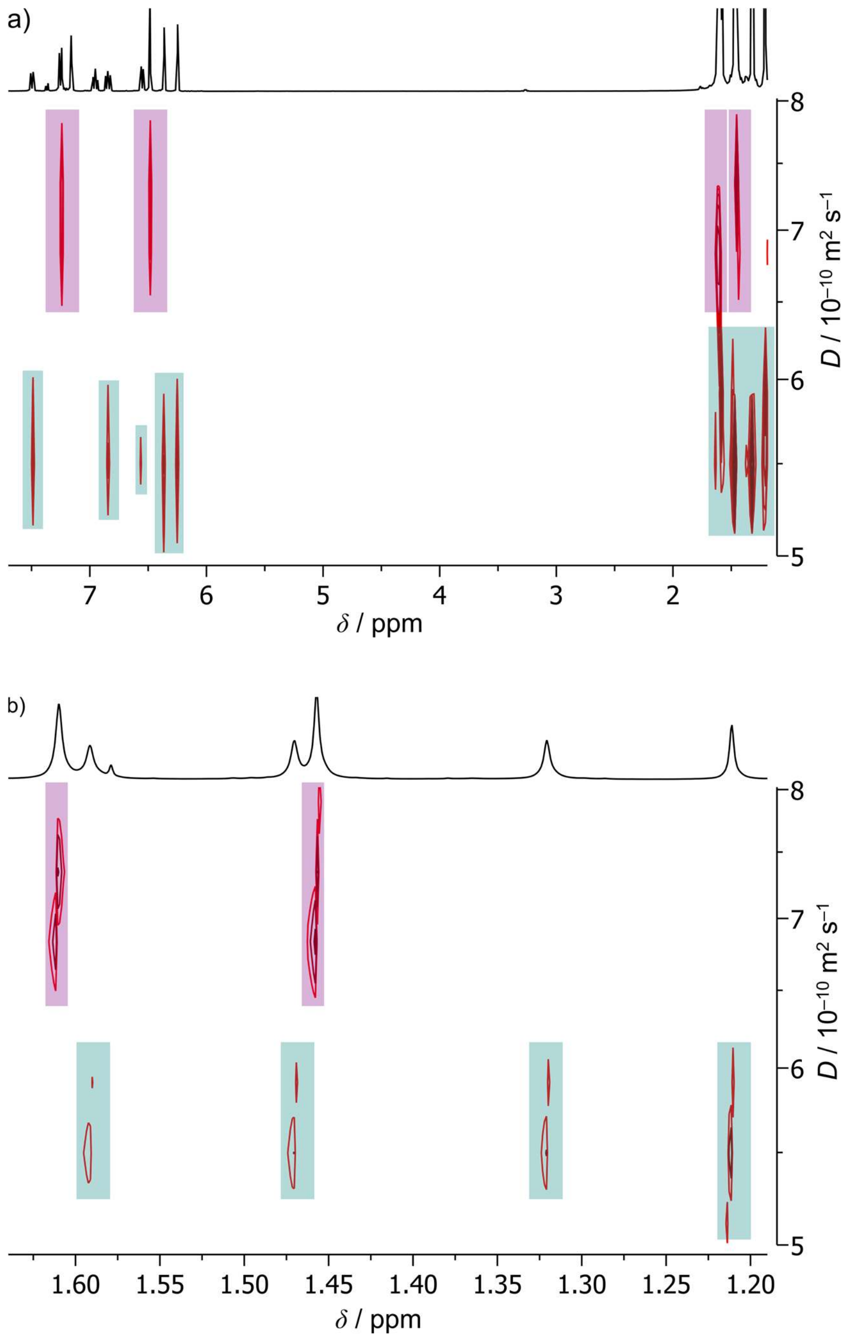

Figure S18. a) ^{1}H NMR DOSY of an equilibrated mixture of **1** and **2** (1:0.57 ratio) in C_6D_6 at 293 K, b) excerpt of the aliphatic region. Correlation peaks of **1** and **2** marked with purple and turquoise bars, respectively

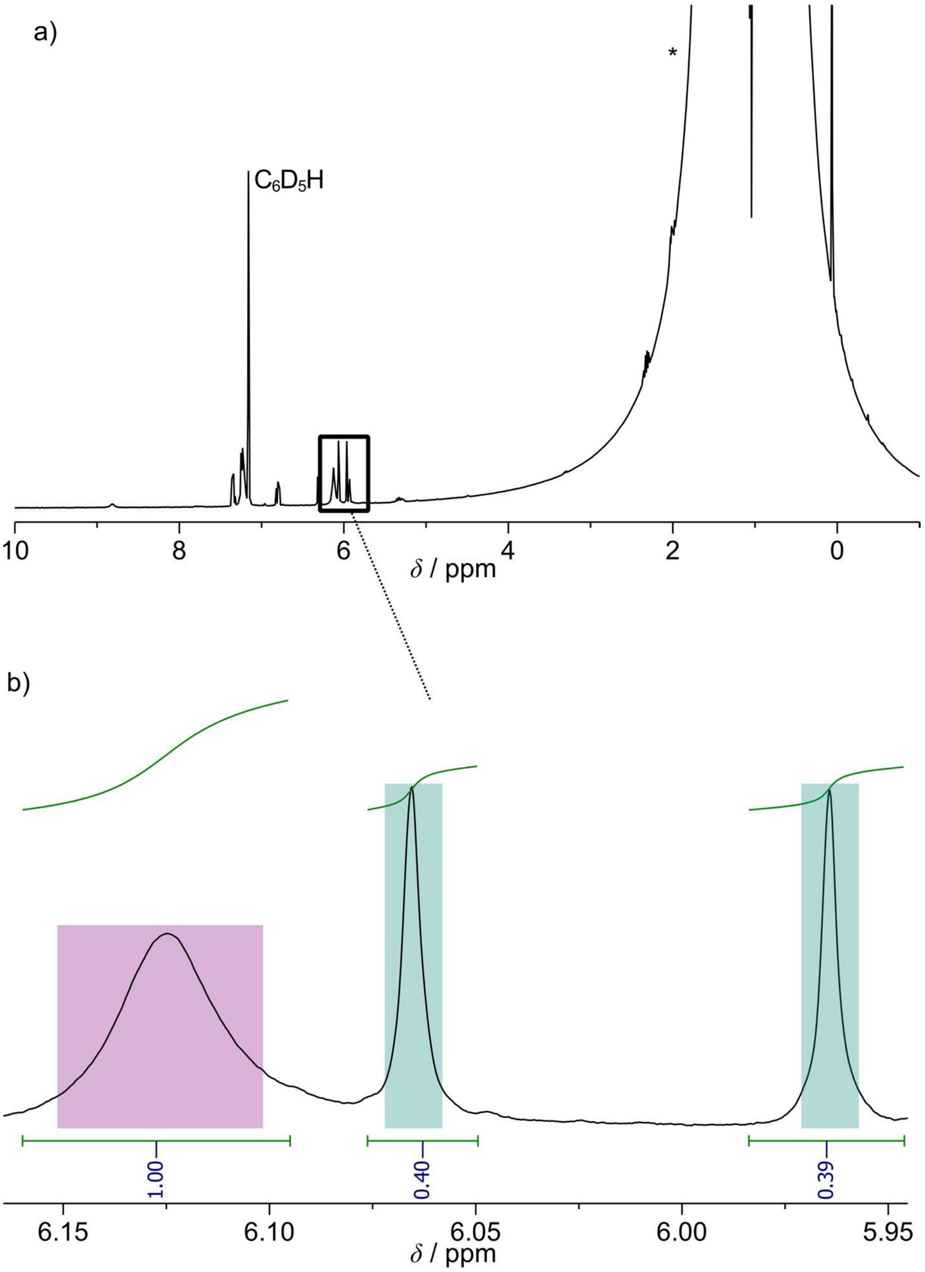

Figure S 19. a) ^{1}H NMR spectrum of a mixture of **1** and **2** in 3-methylpentane (asterisk) with C_6D_6 (for locking) 30 minutes after sample preparation from **1** at 293 K and b) integrated pyrrolide proton resonances of **1** and **2** marked with purple and turquoise bars, respectively.

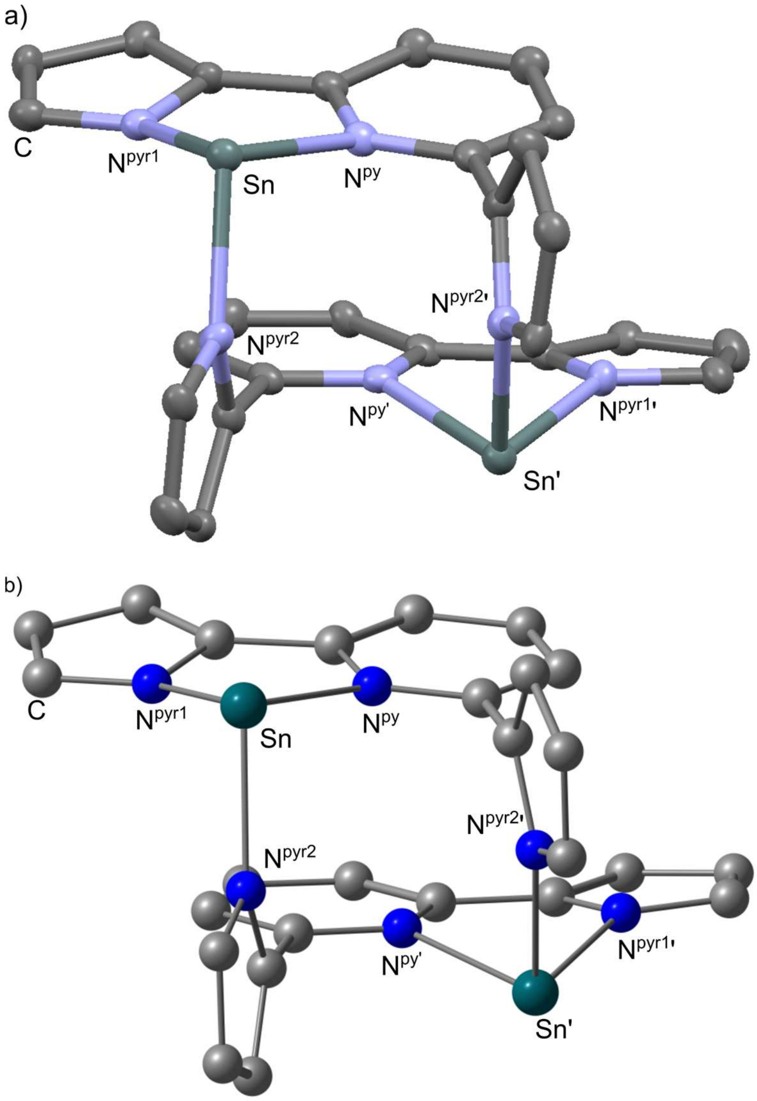

Figure S20. Molecular structure of **2** from a) SC-XRD analysis and b) from DFT calculations, hydrogen atoms and *tert*-butyl substituents omitted, thermal ellipsoids at 50% probability level. CPCM(hexane)-RIJCOSX-B3LYP-D3BJ-ZORA-SARC/J-ZORA-def2-TZVPP/SARC-ZORA-TZVPP(Sn).

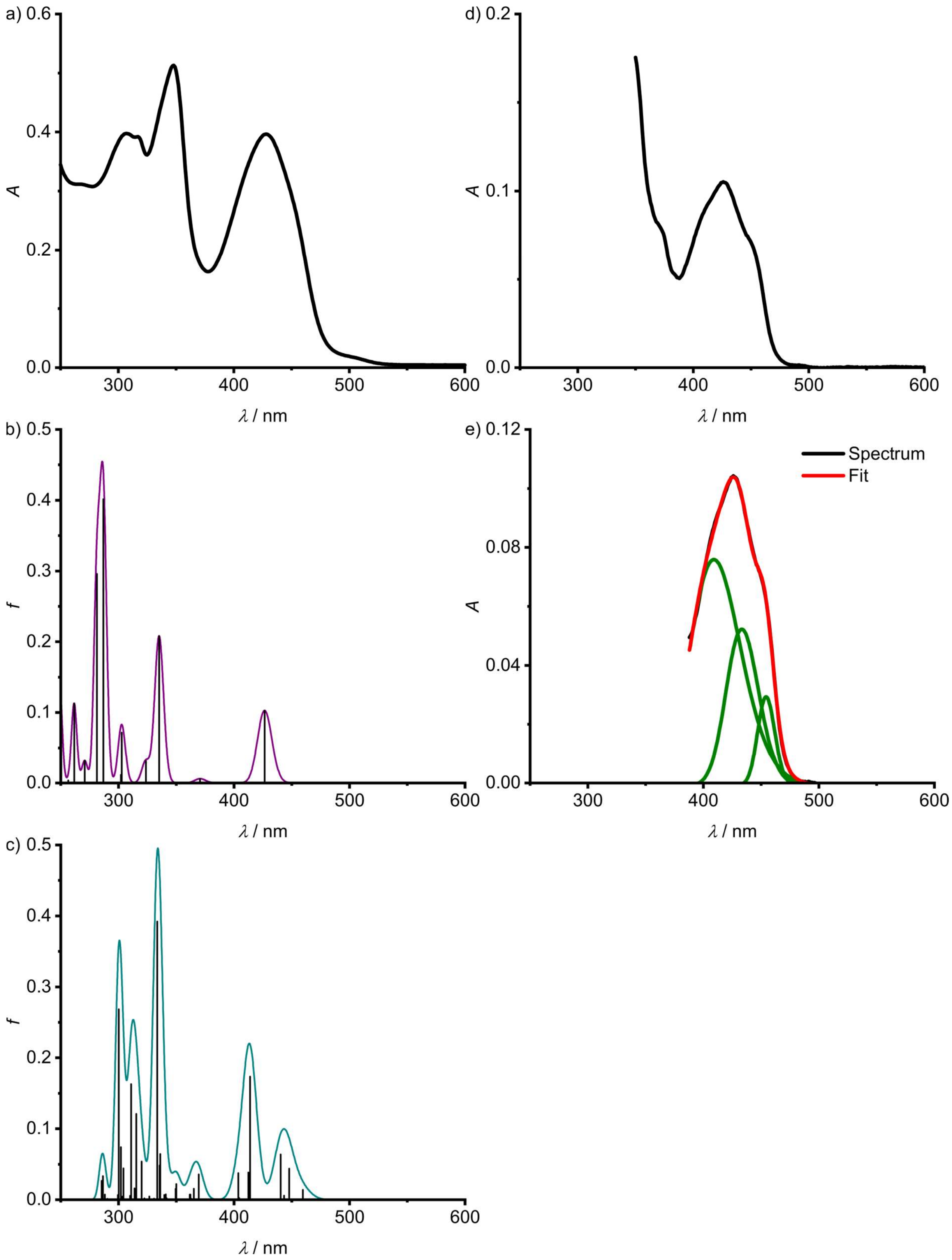

Figure S21. a) Absorption spectrum of a mixture of **1** and **2** in *n*-pentane at 293 K, 30 minutes after sample preparation from **1**. TDDFT calculated oscillator strengths b) of the monomer **1** shifted by +3500 cm^{-1} to higher energies with Voigtian line broadening (FWHM = 800 cm^{-1}) ;and c) of the dimer **2** shifted by –1650 cm^{-1} to lower energies with Voigtian line broadening (FWHM = 800 cm^{-1}); d) absorption spectrum of a mixture of **1** and **2** at 77 K, 25 minutes after sample preparation from **1**; and e) sum fit (red) consisting of three Voigt functions (green) of the low-energy band of the absorption spectrum at 77K. (CPCM(hexane)-RIJCOSX-B3LYP-D3BJ-ZORA-SARC/J-ZORA-def2-TZVPP/SARC-ZORA-TZVPP(Sn)).

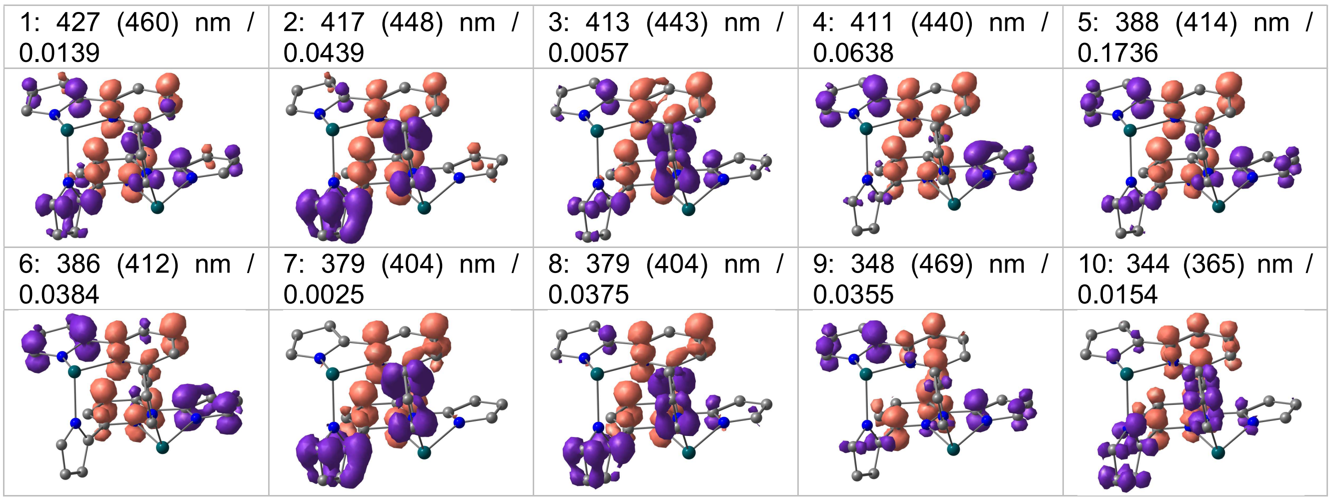

Figure S22. Difference electron densities of the 10 lowest spin-allowed transitions of **2** with the respective wavelength and oscillator strength (purple = electron loss; orange = electron gain) displayed at an isosurface value of 0.003 a.u.. The values in parentheses are shifted by –1650 cm^{-1} to lower energies to fit the absorption spectra depicted in Figure S20. CPCM(hexane)-RIJCOSX-B3LYP-D3BJ-ZORA-SARC/J-ZORA-def2-TZVPP/SARC-ZORA-TZVPP(Sn), hydrogen atoms and *tert*-butyl substituents omitted.

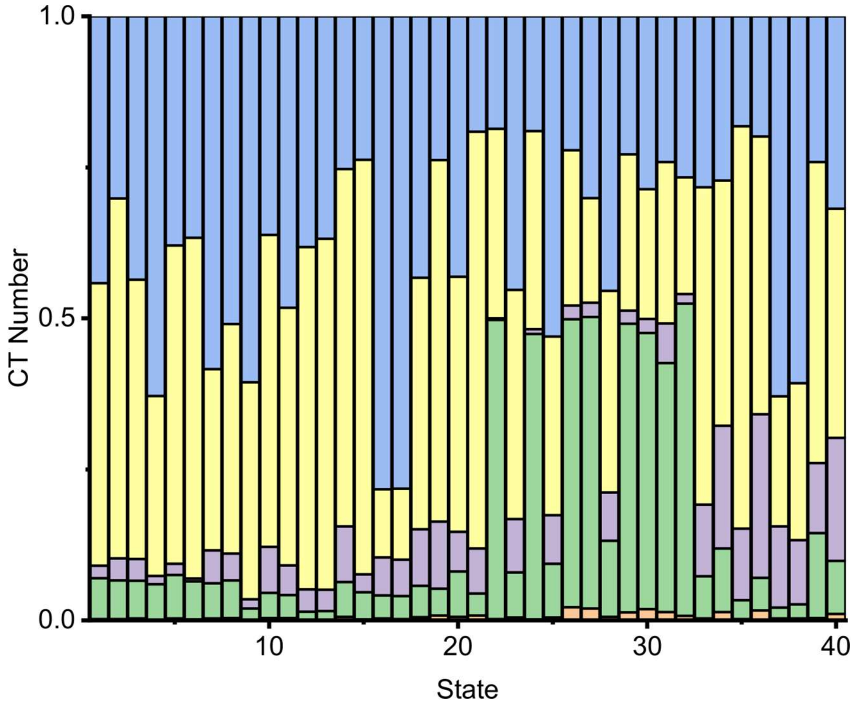

Figure S23. Charge transfer analysis of the 50 lowest spin-allowed transitions of **2** (green: LMCT, yellow: ILCT, purple: MLCT; orange: MC, blue: LL’CT).

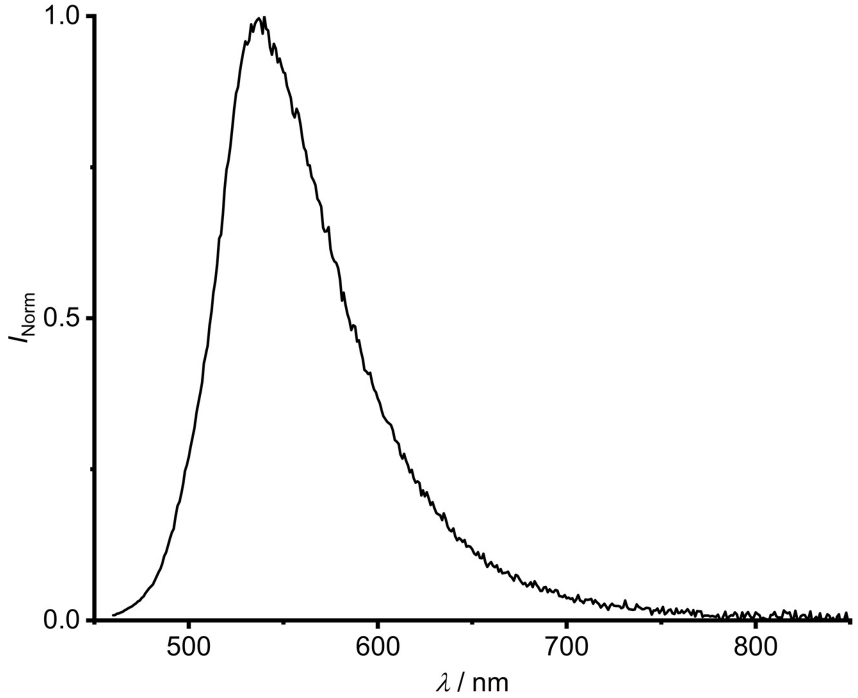

Figure S 24. Emission spectrum of **2** measured from a mixture of **1** and **2** in 3-methylpentane solution at 293 K with λ_p = 540 nm, respectively (λ_{exc} = 450 nm). Only the luminescence of **2** is observed due to the much higher quantum yield.

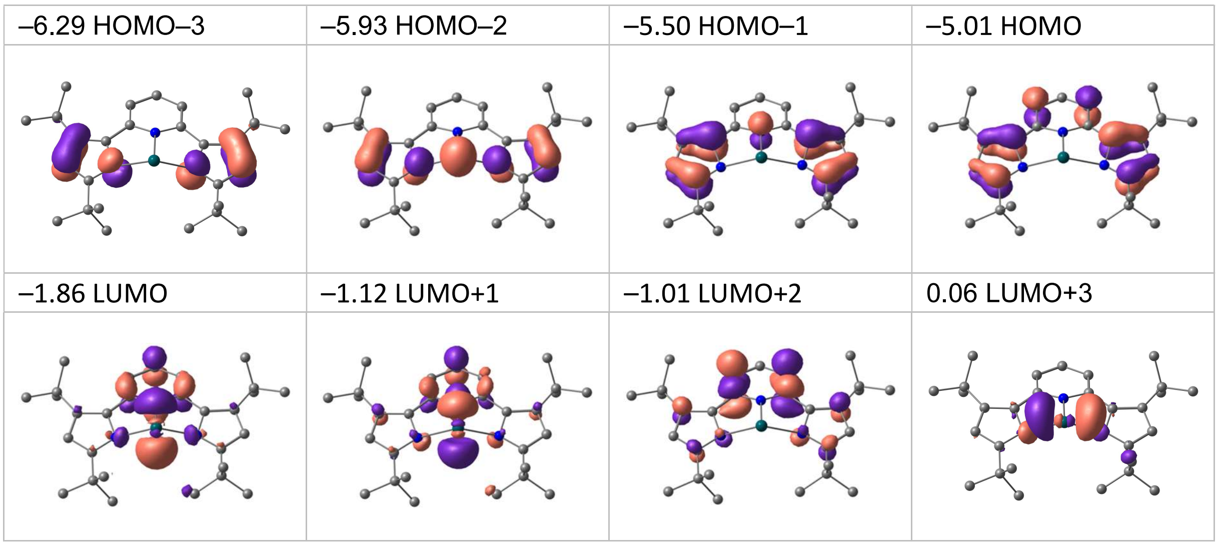

Figure S25. Selected molecular orbitals of **1** with energies given in eV, displayed at an isosurface value of 0.05 a.u.. CPCM(hexane)-RIJCOSX-B3LYP-D3BJ-ZORA-SARC/J-ZORA-def2-TZVPP/SARC-ZORA-TZVPP(Sn), hydrogen atoms omitted.

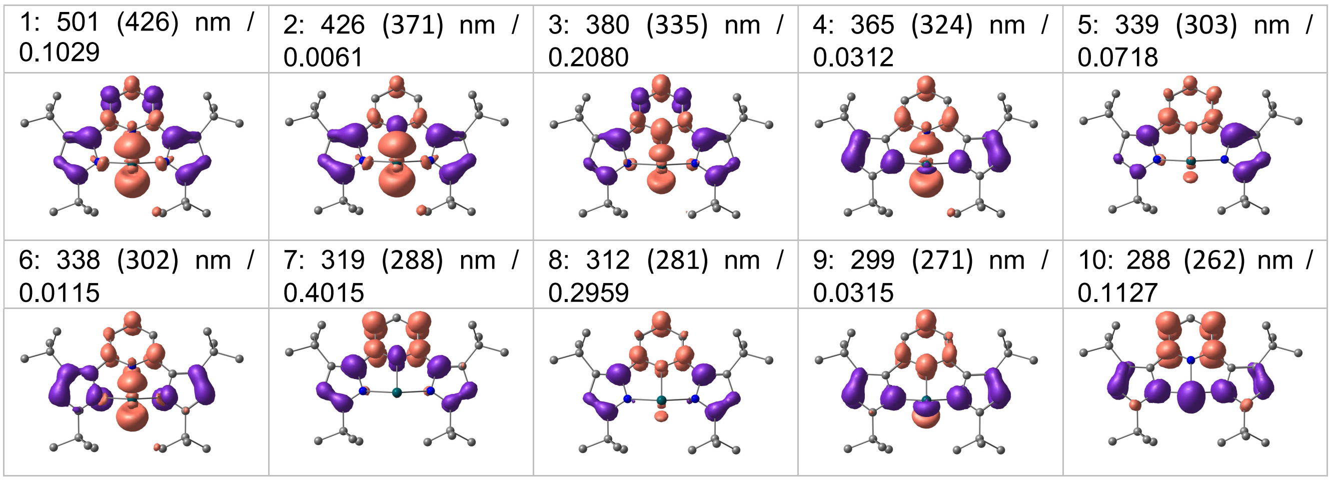

Figure S26. Difference electron densities of the 10 lowest spin-allowed transitions of **1** with the respective wavelength and oscillator strength (purple = electron loss; orange = electron gain) displayed at an isosurface value of 0.003 a.u.. The values in parentheses are shifted by +3500 cm^{-1} to higher energies to fit the absorption spectra depicted in Figure S20. CPCM(hexane)-RIJCOSX-B3LYP-D3BJ-ZORA-SARC/J-ZORA-def2-TZVPP/SARC-ZORA-TZVPP(Sn), hydrogen atoms omitted.

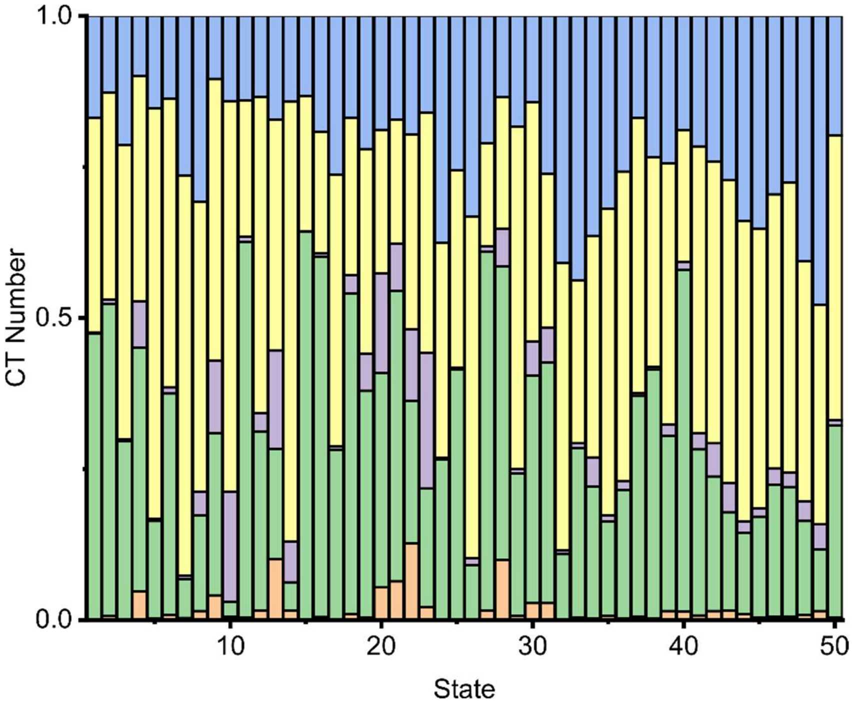

Figure S27. Charge transfer analysis of the 50 lowest spin-allowed transitions of **1** (green: LMCT, yellow: ILCT, purple: MLCT; orange: MC, blue: LC)

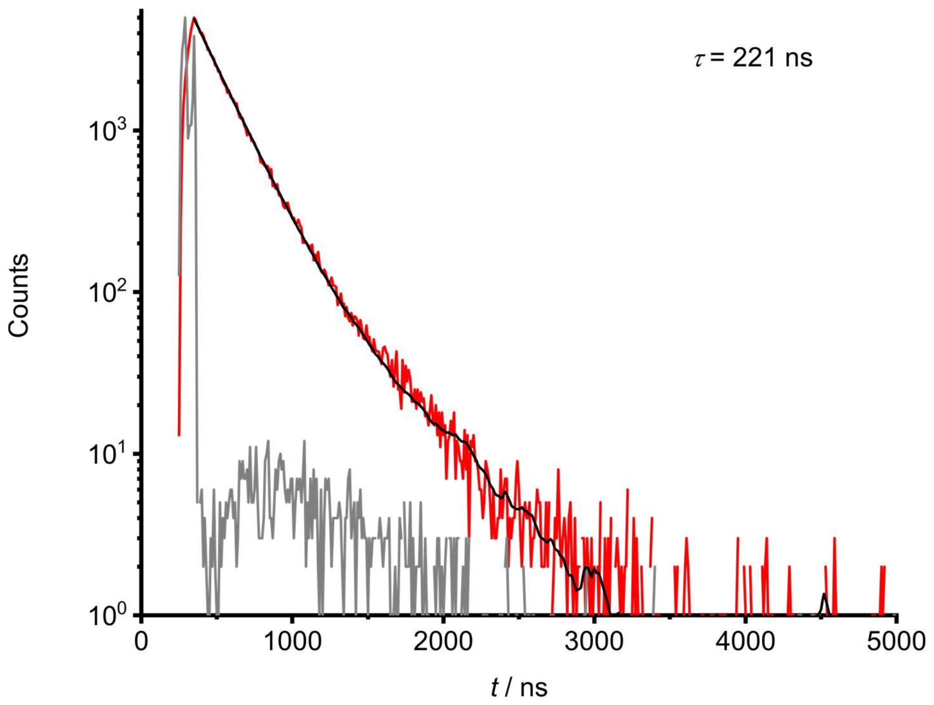

Figure S28. Emission decay curve of **1** in the solid state upon λ_{exc} = 450 nm excitation at λ_{em} = 700 nm at 293 K (red) superimposed with monoexponential reconvolution fit (black) and IRF (gray).

Table S3. Emission properties of **1** in the solid state.

	λ_p / nm	τ_p	Φ_p	k_{obs} / s^{-1}	k_r / s^{-1}	k_{nr} / s^{-1}
293 K	710	221 ns	0.0044 [b]	4.52×10^6	20000	4.5×10^6
77 K	690	128 μs (97%)<br>313 μs (3%) [a]	0.0116 [b]	7.81×10^3 [c]	100 [c]	7.7×10^3 [c]

[a] Relative emission amplitude in parentheses. [b] Photoluminescence quantum yields include the emission band tail in NIR region. [c] Determined for the main component τ_{p1} = 128 μs.

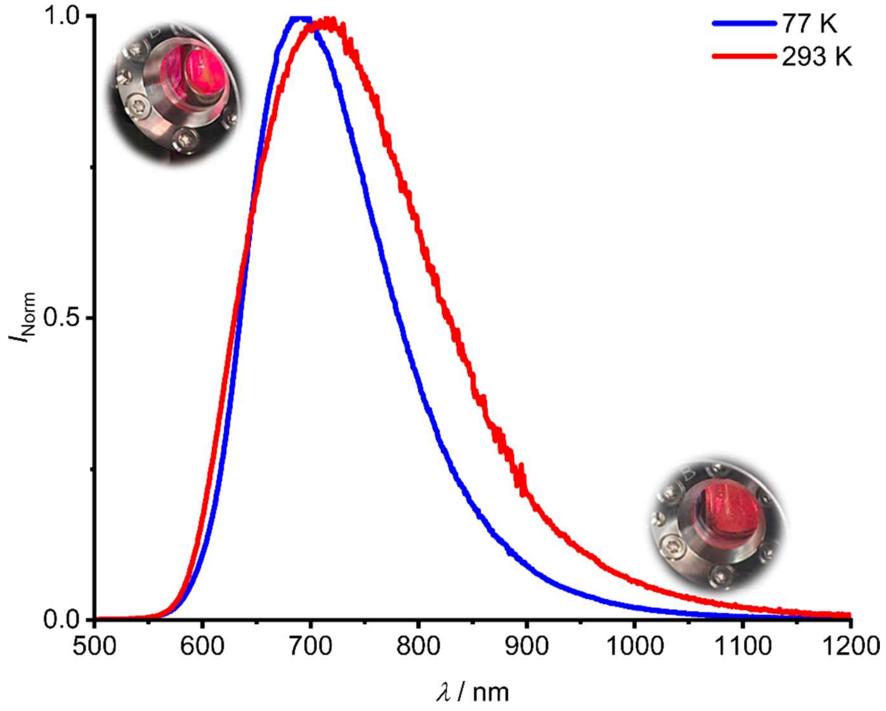

Figure S29. Normalized emission spectra of **1** in the solid state upon λ_{exc} = 450 nm excitation at 77 K (blue) and 293 K (red). Inset: Photographs of the red emission at 77 K (left) and 293 K (right).

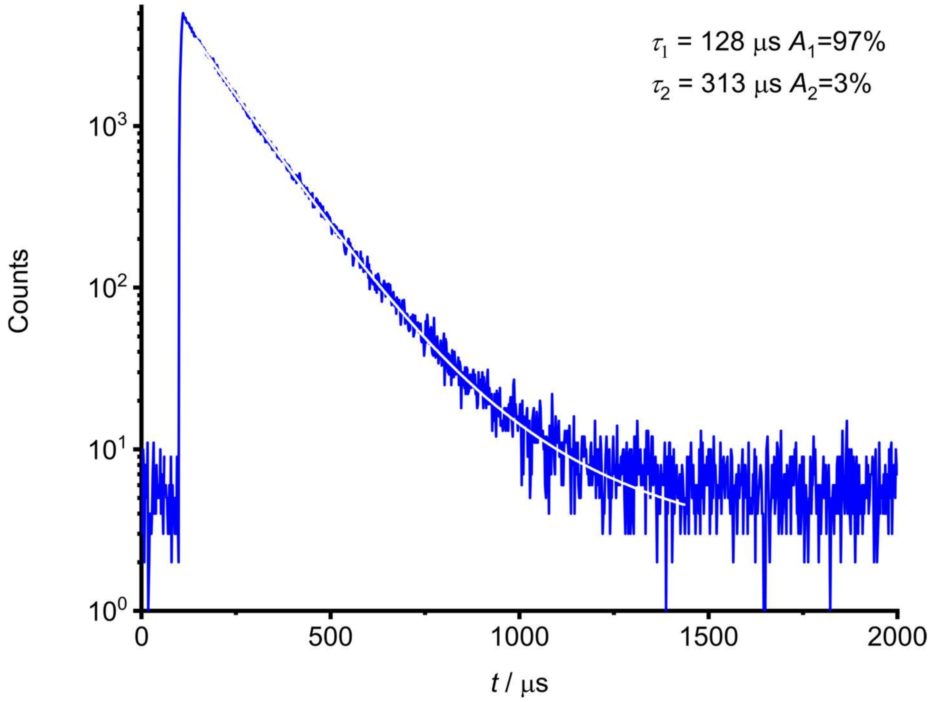

Figure S30. Emission decay curve of **1** in the solid state (blue) upon λ_{exc} = 450 nm excitation at λ_{em} = 700 nm at 77 K superimposed with biexponential fit (white).

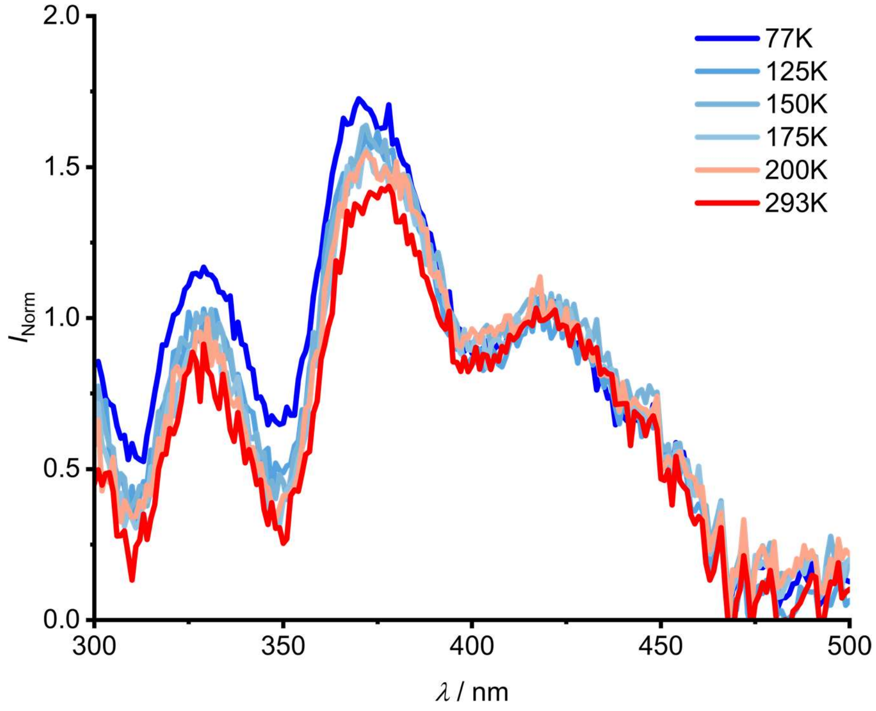

Figure S31. Excitation spectra of **1** at various temperatures in the solid state (λ_{obs} = 700 nm). The high absorbance in the solid state and filter effects lead to distorted spectra in the high energy region of the spectra.

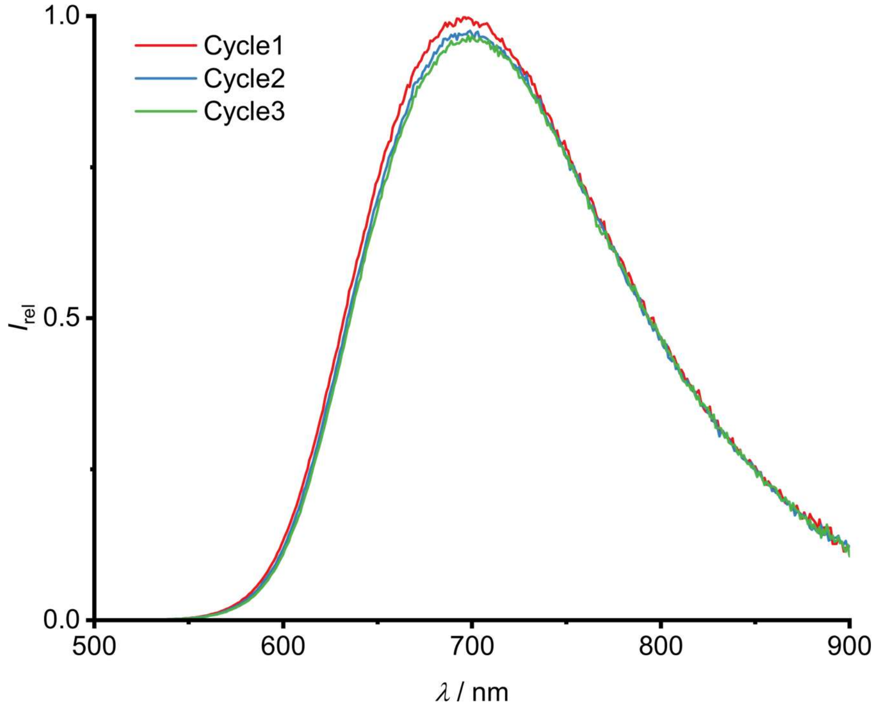

Figure S32. Emission spectra of **1** upon excitation at λ_{exc} = 450 nm in the solid state at 150 K after repeating heating and cooling cycles (77–200 K).

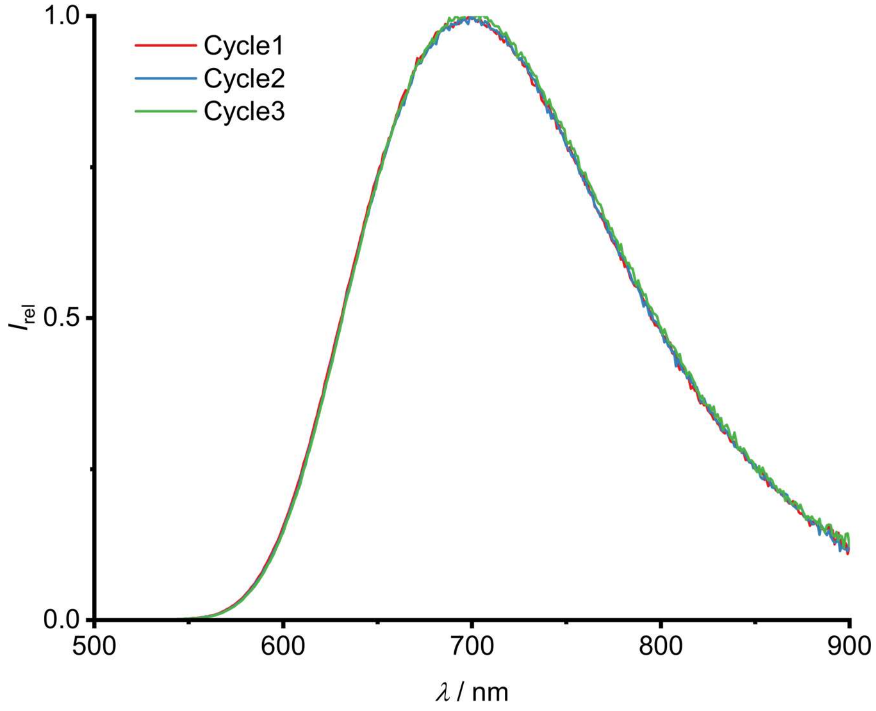

Figure S33. Emission spectra of **1** upon excitation at λ_{exc} = 450 nm in the solid state at 175 K after repeating heating and cooling cycles (77–200 K).

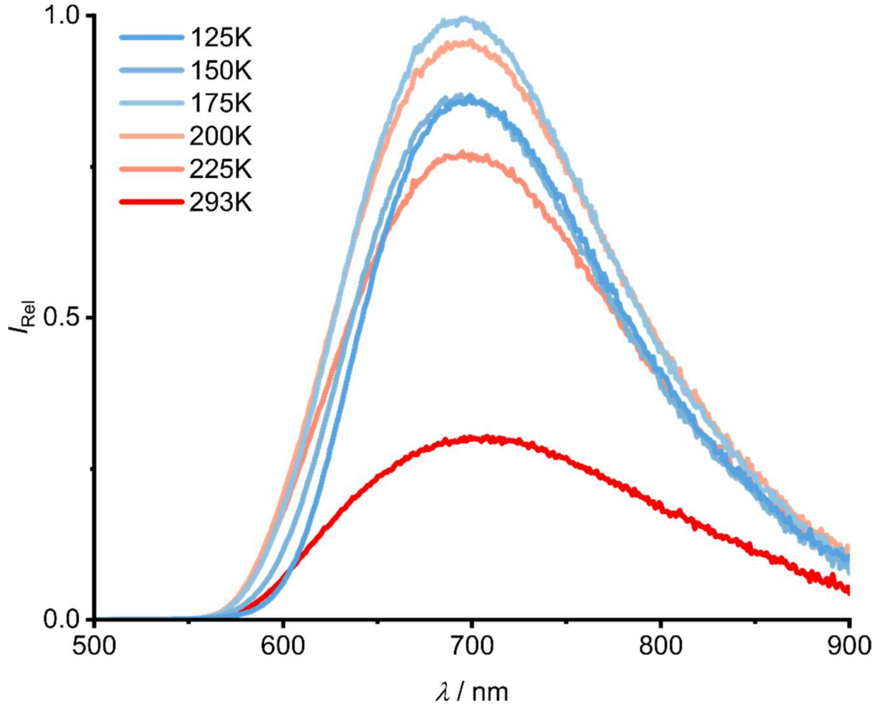

Figure S34. Temperature-dependent emission spectra of **1** upon λ_{exc} = 420 nm excitation in the solid state.

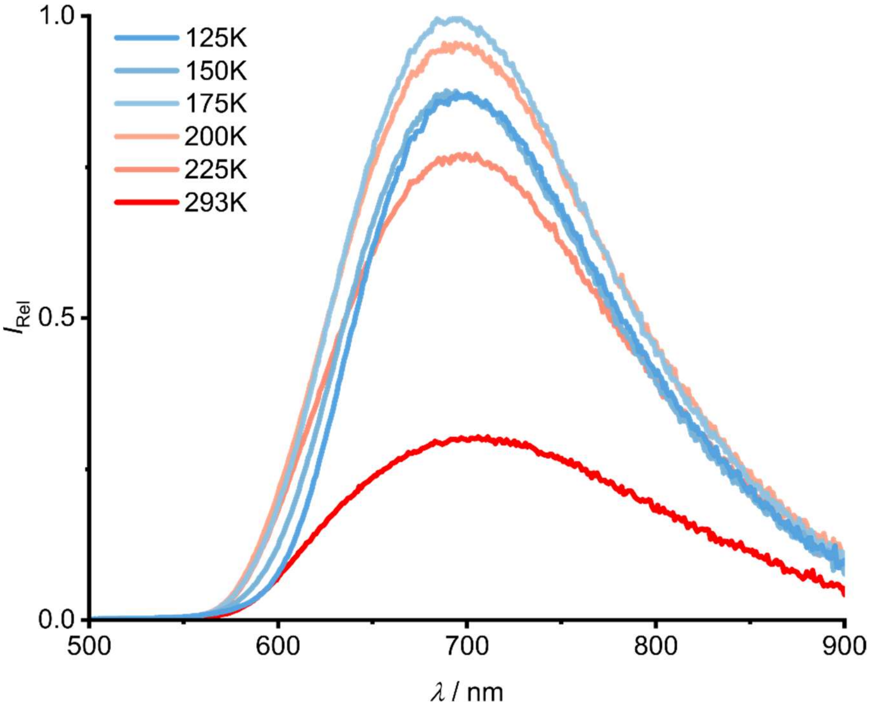

Figure S35. Temperature-dependent emission spectra of **1** upon λ_{exc} = 480 nm excitation in the solid state.

Table S4. Fit parameters for the emission decays k_{obs}, k_{nr} and k_r of solid **1** obtained from Arrhenius fits for given temperature regions.

	$\boldsymbol{k_{obs}}$	**temperature regions / K**
$\boldsymbol{E_{a,1}}$ **/ eV (cm^{-1})**	0.01 (81)	77–100
$\boldsymbol{A_1}$ **/ s^{-1}**	4.40×10^4	
$\boldsymbol{E_{a,2}}$ **/ eV (cm^{-1})**	0.19 (1533)	225–293
$\boldsymbol{A_2}$ **/ s^{-1}**	7.86×10^9	
	$\boldsymbol{k_{nr}}$	
$\boldsymbol{E_{a,1}}$ **/ eV (cm^{-1})**	0.01 (81)	77–100
$\boldsymbol{A_1}$ **/ s^{-1}**	4.44×10^4	
$\boldsymbol{E_{a,2}}$ **/ eV (cm^{-1})**	0.19 (1533)	225–293
$\boldsymbol{A_2}$ **/ s^{-1}**	7.70×10^9	
	$\boldsymbol{k_r}$	
$\boldsymbol{E_{a,r}}$ **/ eV (cm^{-1})**	0.11 (887)	175–293
$\boldsymbol{A_r}$ **/ s^{-1}**	1.52×10^6	

Table S5. Relative integrals of the emission bands of solid **1** at various temperatures with photoluminescence quantum yields $\Phi_{p,rel}$ determined relative to the absolute quantum yield Φ_p at 293 K given in Table S3, emission lifetimes τ_p with respective rate constants k_{obs} and radiative and nonradiative rate constants k_r and k_{nr} determined from $\Phi_{p,rel}$ and k_{obs}.

***T* / K**	**relative integral**	$\Phi_{p,rel}$ [a]	τ_p / µs	k_{obs} / s^{-1}	k_r / s^{-1}	k_{nr} / s^{-1}
293	1.00	0.0044 [b]	0.221	4.52×10^6	20000	4.5×10^6
275	1.29	0.0056	0.385	2.60×10^6	15000	2.6×10^6
250	1.79	0.0078	0.892	1.12×10^6	8700	1.1×10^6
225	2.35	0.010	2.12 [c]	4.73×10^5	4800	4.7×10^5
200	2.80	0.012	5.00 [c]	2.00×10^5	2400	2.0×10^5
175	2.84	0.012	12.0 [c]	8.33×10^4	1000	8.2×10^4
150	2.48	0.011	27.0 [c]	3.70×10^4	400	3.7×10^4
125	2.54	0.011	51.0 [c]	1.96×10^4	220	1.9×10^4
100	3.78	0.017	86.0 [c]	1.16×10^4	190	1.1×10^4
77	5.52	0.024	128 [c]	7.81×10^3	190	7.6×10^3

[a] Relative uncertainty of Φ, k_r ,and k_{nr} is estimated to be ±20%.[b] Absolute photoluminescence quantum yield Φ_p and emission lifetime τ_p see Table S3. [c] Main component (94–98%) of biexponential decay.

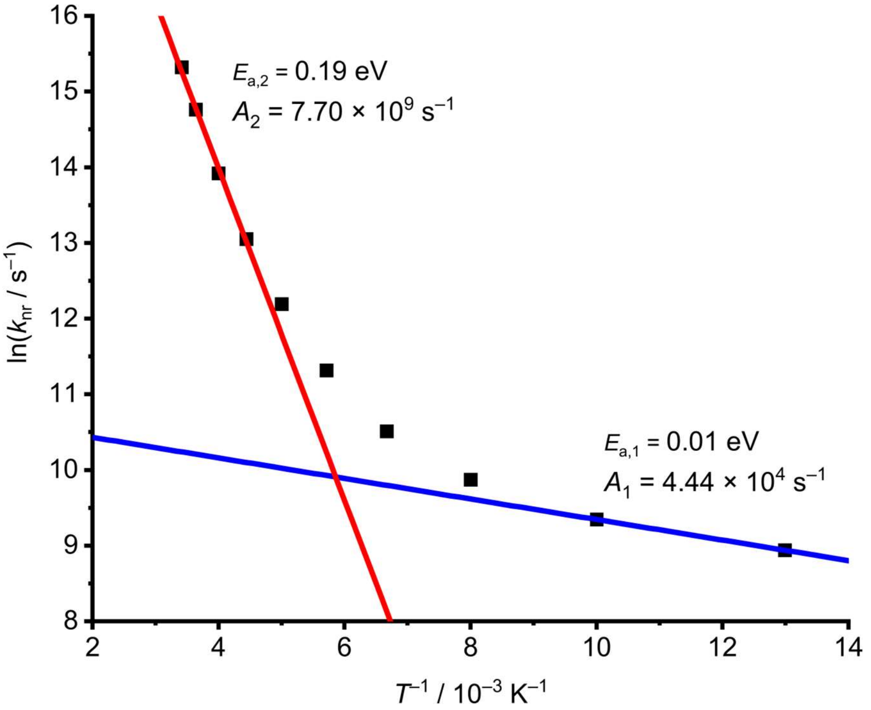

Figure S36. Arrhenius plot of the nonradiative rate constants ($k_{nr}(T)$, T = 77–293 K) of solid **1** with fit parameters obtained from Arrhenius fits in the temperature regions 293–225 and 100–77 K, respectively.

Table S6. SOC-TDDFT calculated SOCCs in cm^{-1} of **1** as square-root of sum of squares of the SOC matrix elements at the FC geometry.

	**T_1**	**T_2**	**T_3**
**S_0**	19	69	163
**S_1**	31	106	72
**S_2**	46	40	2
**S_3**	95	70	88

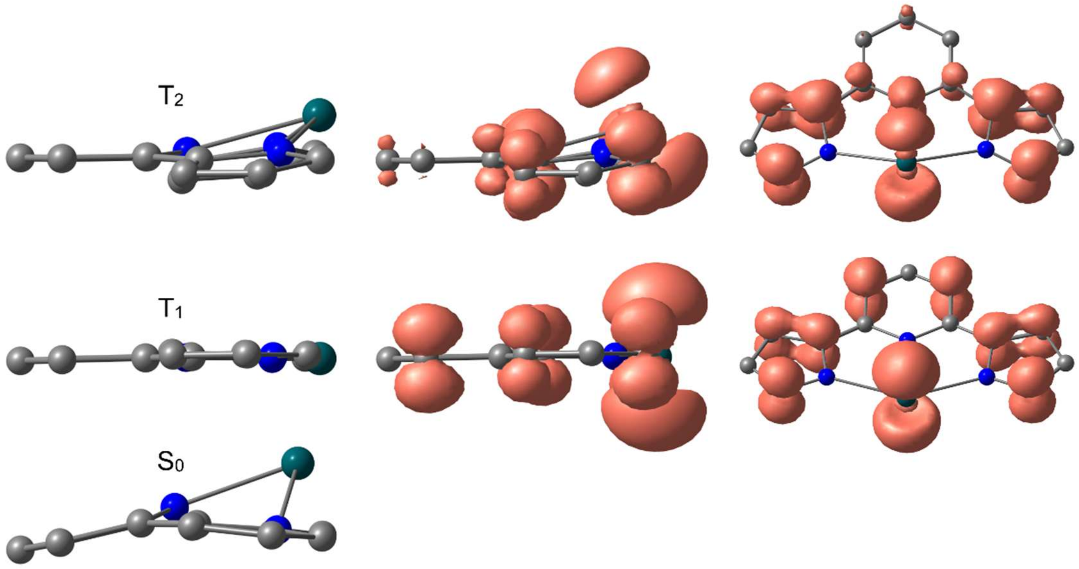

Figure S37. DFT optimized geometries of the singlet ground state S_0 and the triplet states T_1 and T_2 with respective spin density plots from the side and front view of **1**. CPCM(hexane)-RIJCOSX-B3LYP-D3BJ-ZORA-SARC/J-ZORA-def2-TZVPP/SARC-ZORA-TZVPP(Sn), hydrogen atoms and *tert*-butyl substituents omitted (isosurface value: 0.005).

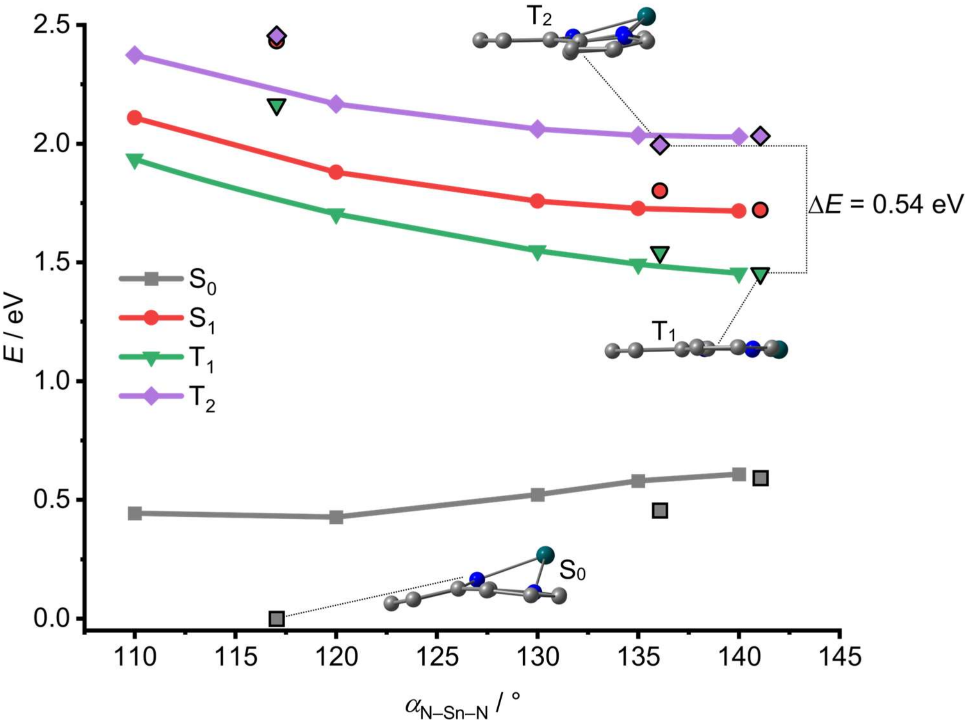

Figure S38. Relaxed potential energy surface scan of **1** as projection along the N^{Pyr1}–Sn–N^{pyr2} bending mode on the T_1 hypersurface with energies from TDDFT calculations for the respective S_0, S_1, T_1 and T_2 hypersurfaces. Symbols with frame indicate the TDDFT calculated energies from the geometry optimized S_0, T_1 and T_2 states with energy difference between the relaxed T_1 and T_2 states indicated. CPCM(hexane)-RIJCOSX-B3LYP-D3BJ-ZORA-SARC/J-ZORA-def2-TZVPP/SARC-ZORA-TZVPP(Sn), hydrogen atoms and *tert*-butyl substituents omitted.

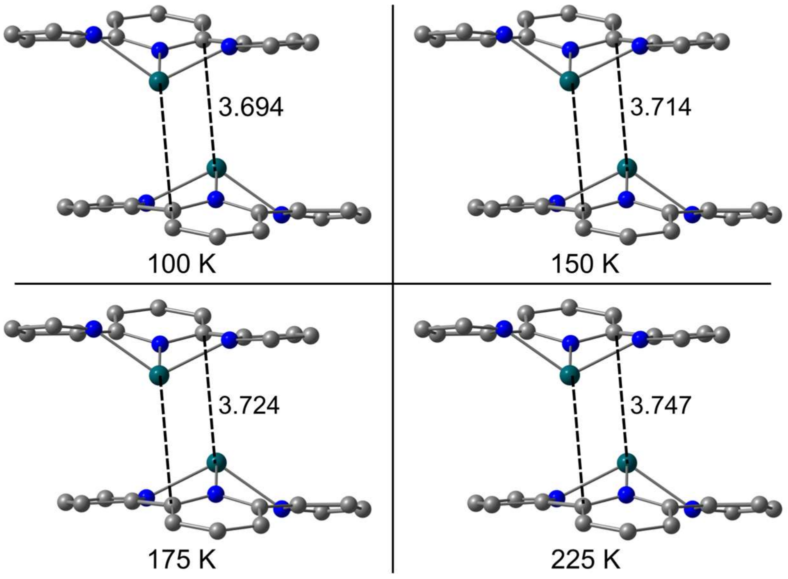

Figure S39. DFT optimized structures (only hydrogen atom positions optimized) of **[1⋯1]^{H}** based on the SC-XRD structures of **1** determined at variable temperatures. *tert*-butyl groups were replaced by hydrogens and only the hydrogen carbon bonds were optimized. CPCM(hexane)-RIJCOSX-B3LYP-D3BJ-ZORA-SARC/J-ZORA-def2-TZVPP/SARC-ZORA-TZVPP(Sn), hydrogen atoms omitted. Sn⋯C17' distances indicated.

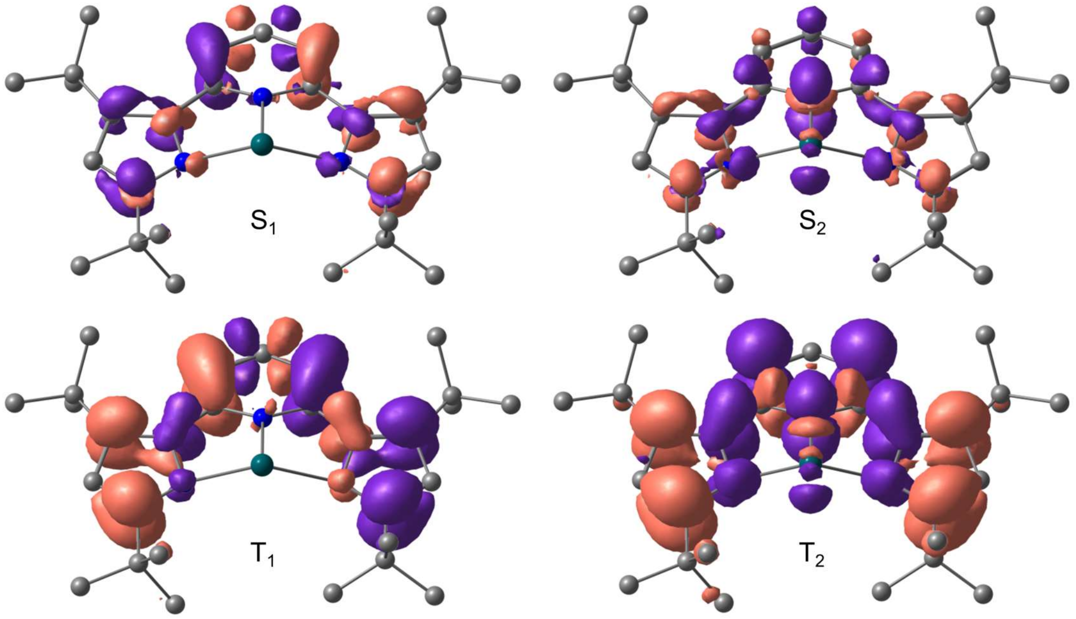

Figure S40. TDDFT calculated transition densities of $S_0 \rightarrow S_1$, $S_0 \rightarrow S_2$, $S_0 \rightarrow T_1$ and $S_0 \rightarrow T_2$ transitions in **1** (isosurface value: 0.001 a.u.).

Table S7. Selected SOC-corrected TDDFT data of **[1···1]^H** based on the SC-XRD structures of **1** at variable temperatures. The electronic states of **[1···1]^H** deriving from the same state in monomeric **1** are denoted $T_{n,+}$/$T_{n,-}$ and $S_{n,+}$/$S_{n,-}$ without distinguishing the M_S spin sublevels of the triplet states (CPCM(hexane)-RIJCOSX-B3LYP-D3BJ-RI-SOMF(1X)-ZORA-SARC/J-ZORA-def2-TZVPP/SARC-ZORA-TZVPP(Sn)).

	**100 K**			**150 K**		
**states**	**energy / cm^{-1}**	**λ / nm**	***f***	**energy / cm^{-1}**	**λ / nm**	***f***
$T_{1,-}$	18577	538.3	0	18584	538.1	0
$T_{1,-}$	18578	538.3	0	18585	538.1	0
$T_{1,-}$	18578	538.3	0	18585	538.1	0
$T_{1,+}$	18593	537.8	1.37×10^{-6}	18607	537.4	2.06×10^{-6}
$T_{1,+}$	18593	537.8	3.470×10^{-7}	18607	537.4	4.63×10^{-7}
$T_{1,+}$	18594	537.8	7.91×10^{-6}	18608	537.4	7.23×10^{-6}
$T_{2,+}$	20862	479.3	4.21×10^{-3}	20897	478.5	5.65×10^{-3}
$T_{2,+}$	20877	479.0	1.48×10^{-6}	20914	478.2	1.49×10^{-6}
$T_{2,+}$	20877	479.0	4.47×10^{-6}	20914	478.2	2.00×10^{-9}
$S_{1,-}$	20893	478.6	0	20914	478.2	6.40×10^{-6}
$T_{2,-}$	20943	477.5	0	20990	476.4	0
$T_{2,-}$	20943	477.5	0	20990	476.4	0
$T_{2,-}$	21074	474.5	0	21078	474.5	0
$S_{1,+}$	21363	468.1	1.51×10^{-1}	21337	468.7	1.51×10^{-1}
	**175 K**			**225 K**		
**states**	**energy / cm^{-1}**	**λ / nm**	***f***	**energy / cm^{-1}**	**λ / nm**	*f*
$T_{1,-}$	18571	538.5	0	18525	539.8	0
$T_{1,-}$	18572	538.5	0	18526	539.8	0
$T_{1,-}$	18572	538.5	0	18526	539.8	0
$T_{1,+}$	18595	537.8	1.50×10^{-6}	18553	539	1.62×10^{-6}
$T_{1,+}$	18595	537.8	2.62×10^{-7}	18553	539	2.28×10^{-7}
$T_{1,+}$	18596	537.8	7.05×10^{-6}	18554	539	7.05×10^{-6}
$T_{2,+}$	20912	478.2	5.74×10^{-3}	20867	479.2	0
$T_{2,+}$	20923	478	1.70×10^{-8}	21005	476.1	1.60×10^{-2}
$T_{2,+}$	20929	477.8	1.68×10^{-6}	21036	475.4	2.37×10^{-6}
$S_{1,-}$	20929	477.8	3.50×10^{-6}	21036	475.4	2.75×10^{-6}
$T_{2,-}$	21002	476.1	0	21100	473.9	0
$T_{2,-}$	21002	476.1	0	21100	473.9	0

**$T_{2,–}$**	21085	474.3	2.00×10^{-9}	21128	473.3	0
**$S_{1,+}$**	21349	468.4	1.51×10^{-1}	21274	470.1	1.29×10^{-1}

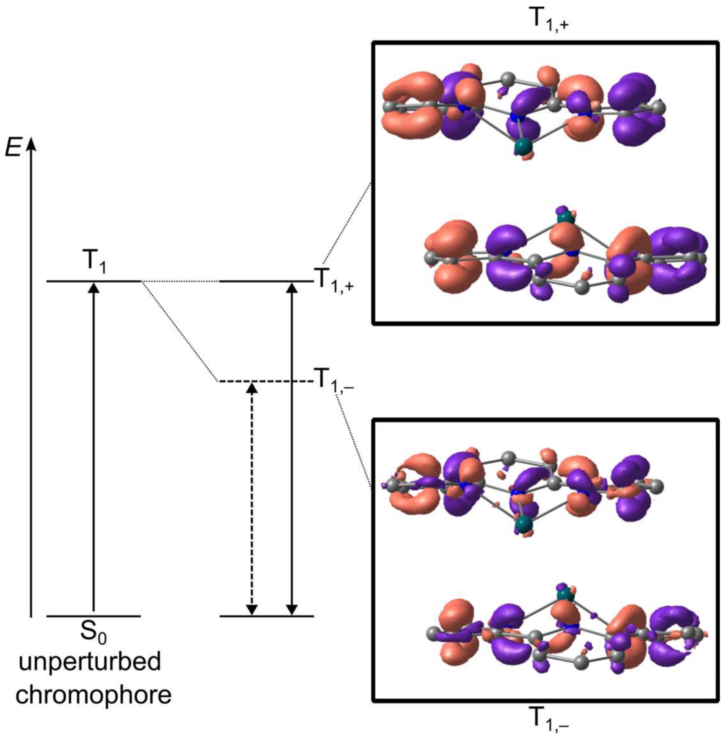

Figure S41. Qualitative energy diagram and SOC-TDDFT calculated $S_0 \rightarrow T_{1,-}/T_{1,+}$ transition densities of **[1···1]^H** derived from the $S_0 \rightarrow T_1$ transition of **1** (isosurface value: 0.001 a.u).

Table S 8. Selected SOC-corrected TDDFT data of **[1···1]^H** based on the SC-XRD structures of **1** at 100 K, calculated with various functionals (CPCM(hexane)-RIJCOSX-D3BJ-RI-SOMF(1X)-ZORA-SARC/J-ZORA-def2-TZVPP/SARC-ZORA-TZVPP(Sn)).

	**B3LYP**				**CAM-B3LYP**		
**states**	**energy / cm^{-1}**	**λ / nm**	***f***	**states**	**energy / cm^{-1}**	**λ / nm**	***f***
$T_{1,-}$	18577	538.3	0	$T_{1,+}$	20162.1	496.0	9.27E-07
$T_{1,-}$	18578	538.3	0	$T_{1,+}$	20162.3	496.0	1.17E-07
$T_{1,-}$	18578	538.3	0	$T_{1,+}$	20162.8	496.0	4.63E-06
$T_{1,+}$	18593	537.8	1.37×10^{-6}	$T_{1,-}$	20170.1	495.8	0
$T_{1,+}$	18593	537.8	3.470×10^{-7}	$T_{1,-}$	20170.3	495.8	0
$T_{1,+}$	18594	537.8	7.91×10^{-6}	$T_{1,-}$	20170.7	495.8	0
$T_{2,+}$	20862	479.3	4.21×10^{-3}	$T_{2,+}$	21397.4	467.3	3.23E-05
$T_{2,+}$	20877	479.0	1.48×10^{-6}	$T_{2,+}$	21397.6	467.3	9.70E-08
$T_{2,+}$	20877	479.0	4.47×10^{-6}	$T_{2,+}$	21398.7	467.3	6.02E-07
$S_{1,-}$	20893	478.6	0	$T_{2,-}$	21398.8	467.3	6.99E-07
$T_{2,-}$	20943	477.5	0	$T_{2,-}$	21399.1	467.3	1.00E-09
$T_{2,-}$	20943	477.5	0	$T_{2,-}$	21399.1	467.3	1.00E-09
$T_{2,-}$	21074	474.5	0	$S_{1,-}$	24806.9	403.1	0.00162
$S_{1,+}$	21363	468.1	1.51×10^{-1}	$S_{1,+}$	25188.8	397.0	0.3184
	**M06-2X**				**TPSSh**		
**states**	**energy / cm^{-1}**	**λ / nm**	***f***		**energy / cm^{-1}**	**λ / nm**	***f***
$T_{1,-}$	14406.9	694.1	0	$T_{1,-}$	18121.4	551.8	0
$T_{1,-}$	14452.8	691.9	0	$T_{1,-}$	18121.5	551.8	0
$T_{1,-}$	14525.5	688.4	0	$T_{1,-}$	18123.7	551.8	0
$T_{1,+}$	14622.5	683.9	2.32E-06	$T_{1,+}$	18154	550.8	1.38E-06
$T_{1,+}$	14624.2	683.8	3.06E-05	$T_{1,+}$	18154.1	550.8	1.79E-07
$T_{1,+}$	14673.5	681.5	3.11E-06	$T_{1,+}$	18157.1	550.7	1.39E-05
$T_{2,-}$	21393.8	467.4	0	$S_{1,-}$	20201.5	495	0
$T_{2,-}$	21394.1	467.4	0	$T_{2,+}$	20268	493.4	0.00374
$T_{2,-}$	21394.4	467.4	0	$T_{2,+}$	20281.5	493.1	3.23E-06
$T_{2,+}$	21432.6	466.6	1.90E-06	$T_{2,+}$	20281.5	493.1	5.65E-06
$T_{2,+}$	21432.6	466.6	4.17E-07	$T_{2,-}$	20442.4	489.2	0
$T_{2,+}$	21432.9	466.6	2.69E-06	$T_{2,-}$	20442.5	489.2	0
$S_{1,-}$	24902.4	401.6	0	$T_{2,-}$	20456	488.9	0

**$S_{1,+}$**	25239.2	396.2	0.31968	**$S_{1,+}$**	20658.1	484.1	0.10435
